# The ARVC-5-associated protein TMEM43 controls mitochondrial energy metabolism by stabilising ER-mitochondrial contact sites

**DOI:** 10.1007/s00018-025-05942-z

**Published:** 2025-11-14

**Authors:** Kai Jürgens, Lena Menzel, Nora Klinke, Lisa Schäper, Leonhard Breitsprecher, Michael Holtmannspötter, Olympia-Ekaterini Psathaki, Stefan Walter, Sandra Ratnavadivel, Anders Malmendal, Heiko Meyer, Hendrik Milting, Achim Paululat

**Affiliations:** 1 Faculty of Biology and Chemistry, Zoology and Developmental Biology, University of Osnabrück, Barbarastraße 11, 49076 Osnabrück, Germany; 2 Integrated Bioimaging Facility iBiOs, Center for Cellular Nanoanalytics Osnabrück (CellNanOs), University of Osnabrück, Barbarastraße 11, 49076 Osnabrück, Germany; 3 Heart- and Diabetescenter NRW, Erich and Hanna Klessmann-Institute for Cardiovascular Research and Development, University Hospital of the Ruhr University Bochum, Georgstr. 11, 32545 Bad Oeynhausen, Germany; 4 Department of Science and Environment, Roskilde University, Universitetsvej 1, 4000 Roskilde, Denmark

**Keywords:** TMEM43, ARVC type 5, Cardiomyopathy, Drosophila model, Cardiogenesis

## Abstract

Arrhythmogenic right ventricular cardiomyopathy type 5 (ARVC-5) is a fully penetrant form of ARVC caused by the missense mutation in the gene *TMEM43*p.S358L. Despite extensive research, the molecular basis underlying the detrimental effects of *TMEM43*p.S358L still needs to be discovered. TMEM43 is a phylogenetically conserved protein. We previously analysed the *Drosophila* homologue (CG8111 or Dmel\Tmem43) to understand the protein's physiological relevance and the mutation p.S358L. *Drosophila* Tmem43 is localised at the ER/SR membrane and interacts with the outer mitochondrial membrane protein Porin/VDAC. This interaction is lost in a Tmem43p.S333L mutant that resembles the human p.S358L mutation. In addition, Tmem43p.S333L caused a breakdown in mitochondrial membrane potential and increased cellular reactive oxygen species, suggesting impaired mitochondrial function as a major pathomechanism. Complementary ultrastructural analyses revealed severe structural defects in the affected mitochondria, including degeneration of the organelles. Highly similar ultrastructural defects were observed in the human right ventricular myocardium of a TMEM43p.S358L trait carrier, suggesting a common molecular basis for the detrimental effects of the mutation in flies and humans. We propose that both the p.S358L mutation in humans and the p.S333L mutation in *Drosophila* impair TMEM43/VDAC interaction, which affects the stability of ER/SR-mitochondrial contact sites and, thus, proper mitochondrial function and oxidative phosphorylation rates. The consequential undersupply of ATP likely results in cardiac cell death and, ultimately, heart failure.

**Supplementary Information:**

The online version contains supplementary material available at 10.1007/s00018-025-05942-z.

## Introduction

In contrast to the clinical course, the cellular and molecular impairments underlying the TMEM43p.S358L related arrhythmogenic cardiomyopathy remain largely unknown. Several animal model systems have been developed to provide insights into the molecular pathology of TMEM43p.S358L. These include knock-out, knock-down, and overexpression models in *Drosophila* [1], zebrafish [2], mice [3], Gu et al., 2021; [4–8], and cultured cells [9, 10]. Jang and colleagues recently studied the TMEM43 function by heterologous expression in CHO-1 cells and postulated that TMEM43 is a non-selective cation channel for Na +, K^+^ and Cs^+^ ions [9]. Gu and colleagues applied cardiac transcriptome analyses to mice to identify genetic correlation, functional enrichment and coexpression networks, validated by a knock-in TMEM43p.S358L mouse model. They found misregulation of several cardiac- and metabolic-related pathways. Based on these data, it was suggested that TMEM43 may also play a role in fat absorption, metabolism, and storage, especially under variable environmental conditions, such as changes in diet [11]. Recently, a nonsense mutation in TMEM43, p.R372X, has been linked to an auditory neuropathy spectrum disorder (ANSD), indicating a TMEM43-related role in cochlea function [12]. Other studies found a correlation between TMEM43 and the cellular DNA damage response [6], NF-κB signalling [8, 13], nuclear stiffness modulation [14], regulation of intercalated disc protein expression [15], and cardiac, small intestine, and metabolic homeostasis [4]. Padrón-Barthe and colleagues provided strong evidence that the mutant protein TMEM43p.S358L shows a partial delocalisation to the cytoplasm, reduced interaction with emerin and β-actin, and artificial activation of glycogen synthase kinase-3β (GSK3β) [5].

We have previously introduced a *Drosophila* model based on the molecular analysis of Dmel\Tmem43 (Flybase gene annotation symbol CG8111), the *Drosophila* homologue of the human TMEM43 protein (Klinke 2022). Throughout this paper, we use Dmel\Tmem43 as an identifier for the *Drosophila* gene/protein to distinguish the *Drosophila* gene/protein from homologues of other species. Our previous work showed that systemic overexpression of the mutant protein, Dmel\Tmem43p.S333L, leads to severe metabolic dysregulation in the animal and, eventually, premature death. However, the heart-specific overexpression of the mutant Dmel\Tmem43p.S333L protein causes arrhythmias and reversible diastolic heart arrest in 5-week-old flies [1]. These characteristics established *Drosophila* as a novel and efficient model to study the molecular basis of TMEM43-related arrhythmogenic cardiomyopathies.

Herein, we utilised affinity purification and immunoprecipitation along with proximity labelling approaches to reveal that Dmel\Tmem43 interacts with Porin, the fly homologue of the vertebrate Voltage-Dependent Anion Channels (VDACs) that localises to the outer mitochondrial membrane. Moreover, Dmel\Tmem43 interacts with the ER/SR-membrane-located calcium pump SERCA and chaperones involved in maintaining ER/SR-mitochondrial contact sites (ERMCSs), such as HRP49. These data support an involvement of Dmel\Tmem43 in the formation or maintenance of ERMCSs in muscle tissue. Consistent with this observation, we found a significantly reduced colocalisation frequency of Dmel\Tmem43p.S333L with Porin, relative to wildtype Dmel\Tmem43 and Porin. In addition, expression of Dmel\Tmem43p.S333L caused a breakdown in mitochondrial membrane potential and increased cellular reactive oxygen species (ROS) levels, suggesting severely impaired mitochondrial function. These physiological impairments were also reflected at the ultrastructural level, with numerous mitochondria exhibiting structural defects or complete degeneration. Mito-ER tether expression revealed significant differences in ERMCS formation, showing impaired, impaired tethering in mutant tissue. Significantly, volumetric 3D-ultrastructural analyses of human right ventricular myocardium obtained from a TMEM43p.S358L carrier revealed highly similar histological defects supporting a common molecular basis for the detrimental effects of the mutation in flies and humans.

Based on these data, we propose that both the p.S333L mutation in Dmel\Tmem43 and the p.S358L mutation in TMEM43 result in an impaired TMEM43/VDAC interaction, which in turn affects the ER/SR-mitochondrial contact sites (ERMCSs). Given the critical roles that ERMCSs play in calcium signalling and facilitation of cellular respiration [16], the disturbed ER/SR-mitochondria interaction should directly affect mitochondrial function and oxidative phosphorylation and eventually result in the accumulation of non-functional mitochondria in cardiomyocytes. The consequential lack of ATP would induce cardiac cell death and replacement by fibroblasts and fat cells. At a certain point, the remaining population of functional cardiomyocytes is no longer sufficient to maintain efficient cardiac function. In an accompanying paper [17], we performed corresponding experiments on mitochondrial function and energy homeostasis in human induced pluripotent stem cells (hiPSCs) and combined our findings with data from human cardiac membrane preparations. The results confirm the transferability of the data obtained in the *Drosophila* model.

## Results

### Dmel\Tmem43 precipitates ER- and mitochondrial proteins in tissue-specific pull-down assays

In previous studies, immunoprecipitation experiments were performed to understand the molecular mechanisms underlying TMEM43p.S358L-related cardiomyopathy and to identify possible interaction partners of the wild-type and mutant TMEM43 proteins. These studies identified, for example, Emerin, Lamin, and other structural nuclear proteins, but also components of the GSK pathway as potential interaction partners of TMEM43 [5, 18]. However, the cell lines used in these studies were, i.e., derived from embryonic kidney cells (HEK cells) or embryonic carcinoma cells (P19 cells), which may not entirely reflect the cardiomyocyte-specific microenvironment or physiological in vivo condition in which TMEM43 naturally acts.

**Fig. 1:**
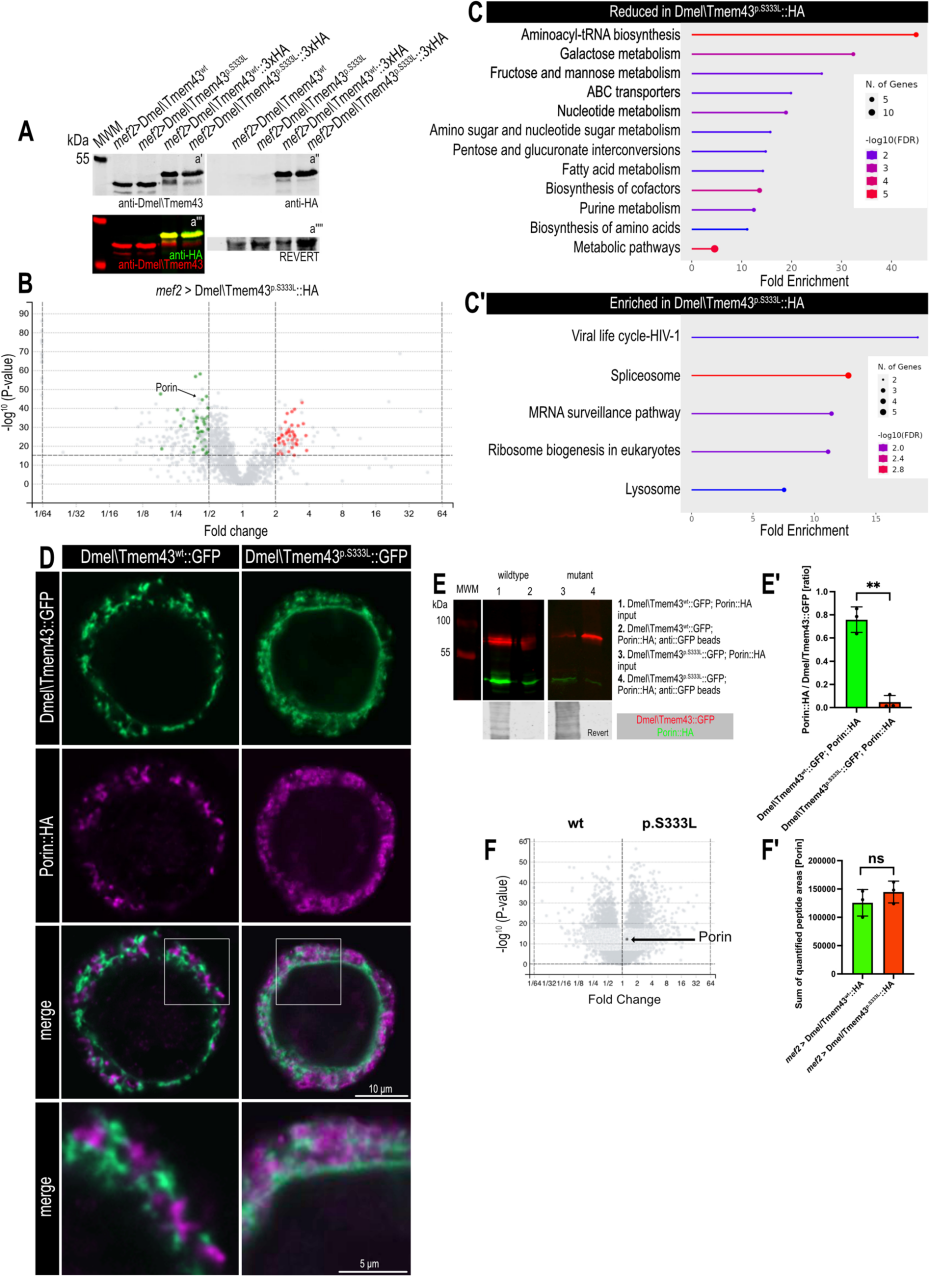
Dmel\Tmem43 interacts with mitochondrial membrane proteins. (**A**) Western blot validation of Dmel\Tmem43wt, Dmel\Tmem43p.S333L, Dmel\Tmem43wt::HA and Dmel\Tmem43p.S333L::HA expression in transgenic flies. Expression was induced in myocytes using *mef2*-Gal4 as a driver. All constructs were stably expressed at comparable levels. (a´) Probed with anti-Dmel\Tmem43, (a´´) probed with anti-HA, (a´´´) Odyssey imaging based fluorescence detection using anti-Dmel\Tmem43 (red) and anti-HA antibodies (green), (a´´´´) REVERT-based total protein stain (loading control). (**B**) Volcano plot depicting the results of pull-down assays using Dmel\Tmem43wt::HA or Dmel\Tmem43p.S333L::HA as bait. Proteins were expressed in 3rd instar larval muscle tissue (*mef2*-Gal4). Green dots represent proteins co-precipitating with Dmel\Tmem43wt::HA, and red dots with Dmel\Tmem43p.S333L::HA. Porin is labelled exemplarily (arrow). Data are based on four individual biological replicates. The corresponding heat map is shown in Supplementary Fig. 1. (**C**) Gene Ontology analyses (KEGG pathways) of proteins exhibiting an increased (C´) or reduced (C) interaction with Dmel\Tmem43p.S333L::HA relative to Dmel\Tmem43wt::HA. (**D**) Immunofluorescence staining of transfected *Sf*21 cells to validate the expression and subcellular localisation of Dmel\Tmem43wt::GFP, Dmel\Tmem43p.S333L::GFP, and Porin::HA. The corresponding cells were also used for co-immunoprecipitation analyses. Cells were stained with anti-GFP (green) and anti-HA antibodies (magenta). Dmel\Tmem43 localises to the ER compartment and Porin to the mitochondrial compartment. (**E**) Co-immunoprecipitation analysis of Dmel\Tmem43wt::GFP or p.S333L::GFP with Porin::HA. A representative blot is depicted. Revert-based total protein stain is shown as a loading control. (E´) Quantification of (E). Dmel\Tmem43wt::GFP; Porin::HA lysates, precipitated with anti-GFP beads, contain significantly more Porin::HA, relative to Dmel\Tmem43p.S333L::GFP; Porin::HA lysates (unpaired t-test, p** < 0.01). All values are normalised to the corresponding amounts of precipitated Dmel\Tmem43. (**F**) Volcano plot of whole proteome preparations isolated from *mef2* > Dmel\Tmem43wt::HA or *mef2* > Dmel\Tmem43p.S333L::HA. Porin amount is indicated by an arrow. (F') Sum of quantified peptide areas for Porin protein levels show no significant changes between the individual genotypes (unpaired t-test). ns = not significant

Therefore, we utilised our TMEM43 *Drosophila* model and established transgenic flies carrying either UAS-Dmel\Tmem43wt::HA or the mutant UAS-Dmel\Tmem43p.S333L::HA as individual fusion proteins suitable for pull-down experiments. Tissue-specific expression was induced in cardiac and somatic muscles with *mef2-*Gal4 as a driver. Proper expression and stability of the fusion proteins were confirmed by Western blot (Fig. 1A). For affinity purification, total protein extracts from third-instar larvae were collected and used for anti-HA-based immunoprecipitation. Isolated proteins were identified by mass spectrometry. In a previous study, we have shown that these constructs are expressed in cardiac cells and are localised in the SR membrane, where they cause no (Dmel\Tmem43wt, control) or severe cardiac arrhythmias and reversible cardiac arrest (Dmel\Tmem43p.S333L, mutant) [1].

As a result, we found numerous proteins that co-immunoprecipitated in equal amounts with both Dmel\Tmem43wt::HA and Dmel\Tmem43p.S333L::HA. In addition, we identified 83 differentially interacting proteins; 39 of those were enriched in the Dmel\Tmem43wt fraction, and 44 were more abundant in the Dmel\Tmem43p.S333L fraction (Fig. 1B, Supplementary Table 1). GO-term analyses of the proteins exhibiting reduced interaction with mutated Dmel\Tmem43 revealed, among others, pathways associated with fatty acid and energy metabolism (Fig. 1C). Interestingly, many underlying proteins are known to be present in mitochondria (Supplementary Table 1). Dmel\Tmem43 does not localise to mitochondria (Supplementary Fig. 3), so its co-precipitation with mitochondrial proteins indicates interaction at ER/SR-mitochondrial contact sites. Among the mitochondrial proteins, Porin was identified as having very high significance. Porin is the fly homologue of the vertebrate voltage-dependent anion channels (VADCs) that localise in the outer mitochondrial membrane (OMM). Significantly, Porin interaction was only observed with wild-type Dmel\Tmem43, while it was absent when the mutant protein was used as bait (Fig. 1B, Supplementary Table 1).

### Porin interacts with Dmel\Tmem43

To further validate Porin as an interacting partner of Dmel\Tmem43, Porin::HA and Dmel\Tmem43wt::GFP fusion proteins were co-expressed in *Sf*21 cells. As expected, Dmel\Tmem43wt::GFP localised in a subcellular pattern reflecting the ER, while Porin::HA localises to mitochondria (Fig. 1D). Co-immunoprecipitation analyses with anti-GFP coupled magnetic beads confirmed the interaction between the proteins. In wildtype samples, significantly more Porin::HA protein amounts co-precipitated with Dmel\Tmem43, relative to mutant samples (Fig. 1E, E´). In order to exclude differences in basal Porin levels, we also measured relative protein abundance in corresponding animals by quantitative mass spectrometry (Fig. 1F, F'). No significant differences were observed for the individual genotypes.

These data confirm that Porin is an interacting partner of Dmel\Tmem43. Moreover, as already observed in transgenic animals, the mutant form Dmel\Tmem43^p.S333L^ exhibited significantly reduced affinity to Porin relative to the wild-type protein.

### Association of Dmel\Tmem43 with ER/SR-mitochondrial contact sites

Interaction of Dmel\Tmem43 with Porin requires physical proximity within the cell. Dmel\Tmem43 localises to the ER/SR membrane, while Porin is an abundant factor of the outer mitochondrial membrane, close spatial proximity can only occur at ER/SR-mitochondrial membrane contact sites (ERMCSs). To verify whether Dmel\Tmem43 localises to ERMCSs, we applied three experimental strategies: (i) a biotinylation-based proximity labelling approach to investigate the physical microenvironment of Dmel\Tmem43 in more detail, (ii) DNA-PAINT-based super-resolution microscopy to quantitatively analyse the subcellular localisation and possible interactions of wild-type and mutant Dmel\Tmem43 at ERMCSs, and (iii) we expressed a well-characterised ERMCS tether construct to quantify the colocalization of Dmel\Tmem43 constructs with the mito-ER tether at ERMCS, [19–21].

### Biotinylation-based proximity labeling substantiates an interaction between Dmel\Tmem43 and Porin

**Fig. 2:**
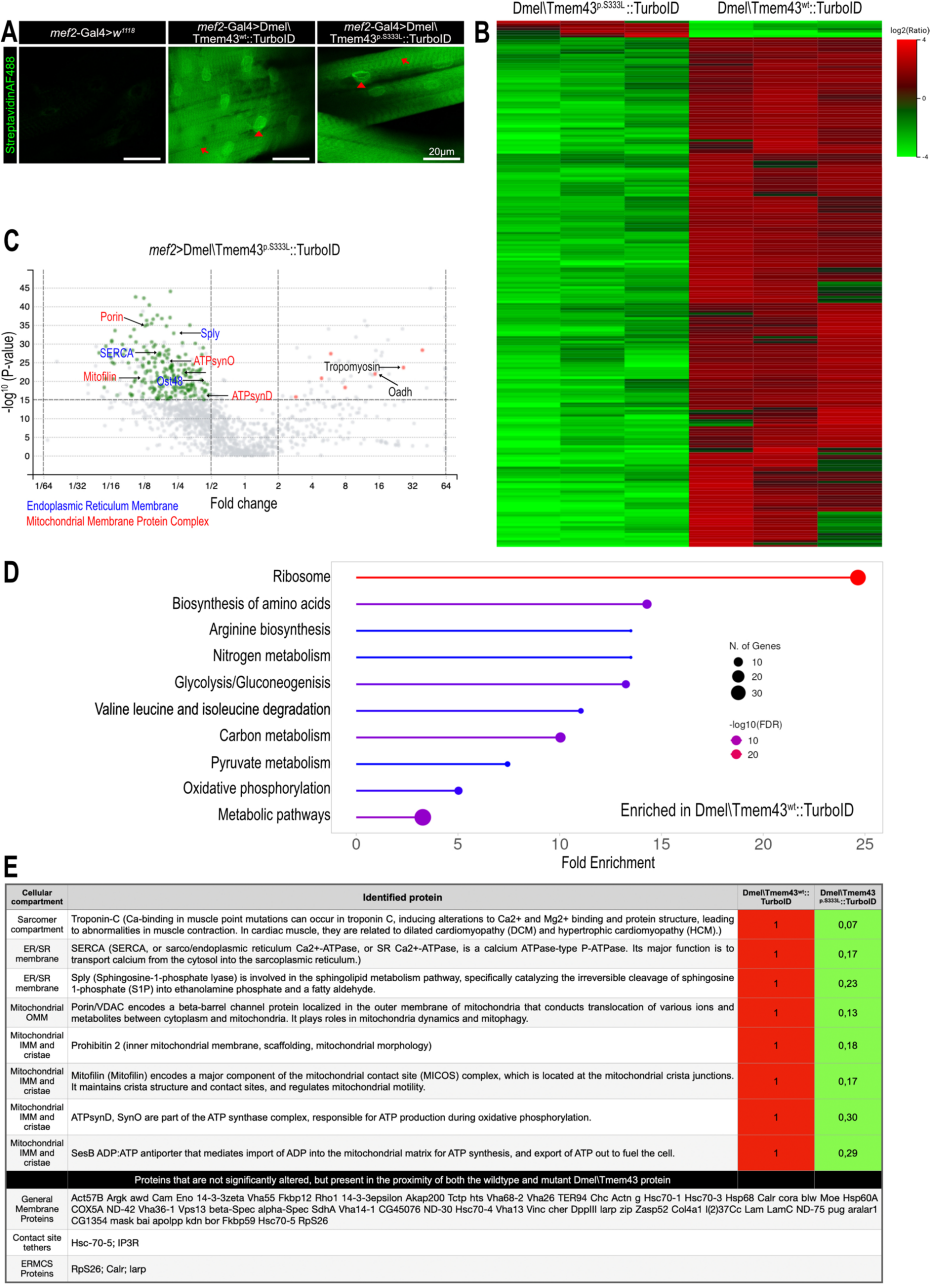
TURBO-ID-based biotin proximity labelling indicates involvement of Dmel\Tmem43 in ER/SR-mitochondrial interaction. (**A**) To validate the expression and subcellular localisation of the Dmel\Tmem43wt::TurboID and Dmel\Tmem43p.S333L::TurboID constructs in transgenic flies, respective tissues were stained with streptavidinAF488. Expression was induced in a muscle-specific manner (*mef2*-Gal4). The Streptavidin signal is present in the SR (arrowheads) and nuclear envelope (arrows) in muscle cells, indicating a wild-type-like subcellular localisation for both constructs. (**B**) Heatmap depicting the results of biotin proximity labelling assays using Dmel\Tmem43wt::TurboID or Dmel\Tmem43p.S333L::TurboID as individual constructs. Proteins were expressed in 3rd instar larval muscle tissue (*mef2*-Gal4). (**C**) Volcano plot depicting the results of biotin proximity labelling assays using Dmel\Tmem43wt::TurboID or Dmel\Tmem43p.S333L::TurboID as individual constructs. Proteins were expressed in 3rd instar larval muscle tissue (*mef2*-Gal4). The identity of selected proteins is indicated (red = proteins with increased abundance in Dmel\Tmem43p.S333L::TurboID animals; green = proteins with increased abundance in Dmel\Tmem43wt::TurboID animals). Data are based on three individual biological replicates. (**D**) Gene Ontology analysis (KEGG pathways) of proteins with reduced abundance in Dmel\Tmem43p.S333L::TurboID animals, relative to Dmel\Tmem43wt::TurboID animals. (E, upper panel) List of selected proteins exhibiting reduced abundance in Dmel\Tmem43p.S333L samples, relative to Dmel\Tmem43wt samples, along with the individual depletion factors. (E, lower panel) Selected proteins that are in close proximity to the two constructs but whose abundance does not change significantly between them

We utilised TurboID biotinylation-based proximity labelling to analyse the Dmel\Tmem43 microenvironment in vivo in transgenic *Drosophila* [22]. The TurboID biotin ligase [23] was fused to either Dmel\Tmem43wt or to Dmel\Tmem43p.S333L to generate Dmel\Tmem43wt::TurboID and Dmel\Tmem43p.S333L::TurboID, respectively (Fig. 2A). Both constructs were expressed in somatic muscle cells using *mef2*-Gal4 as a driver. Muscle integrity was confirmed by Phalloidin stainings (Supplementary Fig. 2A). Expression of functional biotin ligase fusion proteins in the transgenic flies was confirmed by whole-mount stainings of dissected larvae with streptavidin-AlexaFluor488. SR/ER and the nuclear envelope were labelled for both constructs in somatic muscles and heart tissue, indicating proper expression of the respective fusion proteins and considerable biotin ligase activity (Fig. 2A, Supplementary Fig. 2B). After streptavidin-based purification, biotinylated proteins from flies of both genotypes were identified by mass spectrometry (Fig. 2B and C). GO term analyses (KEGG) of the proteins enriched in the Dmel\Tmem43wt samples again identified processes involved in mitochondrial energy metabolism, including oxidative phosphorylation (Fig. 2D), thus further supporting the data obtained in the pull-down assays (Fig. 1C). On the other hand, processes enriched in Dmel\Tmem43p.S333L::TurboID samples, compared to Dmel\Tmem43wt::TurboID samples, were restricted to peroxidase and peroxiredoxin activity, indicating a need to dispose of reactive oxygen species in the corresponding tissues (Supplementary Fig. 2C). A complete list of the proteins identified by proximity biotinylation is depicted in (Supplementary Table 2).

In line with the co-immunoprecipitation data (Fig. 1B), Porin was detected among the proteins exhibiting the highest enrichment factors in the biotinylation assay (Fig. 2C). Significantly, based on the relative amounts of biotinylated protein, wild-type Dmel\Tmem43 interacted approximately eight times stronger with Porin than the mutant form, which provides further evidence that the mutation in Dmel\Tmem43p.S333L severely affects the protein´s ability to interact with Porin. In addition to Porin, we identified other mitochondrial proteins, including Prohibitin2, a scaffolding protein; Mitofilin, a component of the mitochondrial contact site and cristae organising system (MICOS); and ATPsynD, a subunit of the mitochondrial F-type ATPase involved in ATP production via oxidative phosphorylation. Because the OXPHOS machinery is present in the inner mitochondrial membrane that should be inaccessible to Dmel\Tmem43-mediated biotinylation, part of the identified proteins may be enriched due to co-isolation of whole mitochondria-derived vesicles.

On the other hand, we also identified characteristic ER/SR proteins as significantly enriched in Dmel\Tmem43wt::TurboID samples relative to Dmel\Tmem43p.S333L::TurboID samples. These included the Ca^2+^-pump SERCA and Sphingosine-1-phosphatase lyase (Sply), a significant factor controlling sphingolipid metabolism, thus supporting the validity and specificity of our experimental approach. A set of proteins significantly enriched in the Dmel\Tmem43wt::TurboID preparations with relevance to ERMCS formation or mitochondrial energy metabolism is depicted in (Fig. 2E).

### DNA-PAINT-based super-resolution microscopy confirms colocalisation of Dmel\Tmem43 with Porin at ER/SR-mitochondrial contact sites

**Fig. 3:**
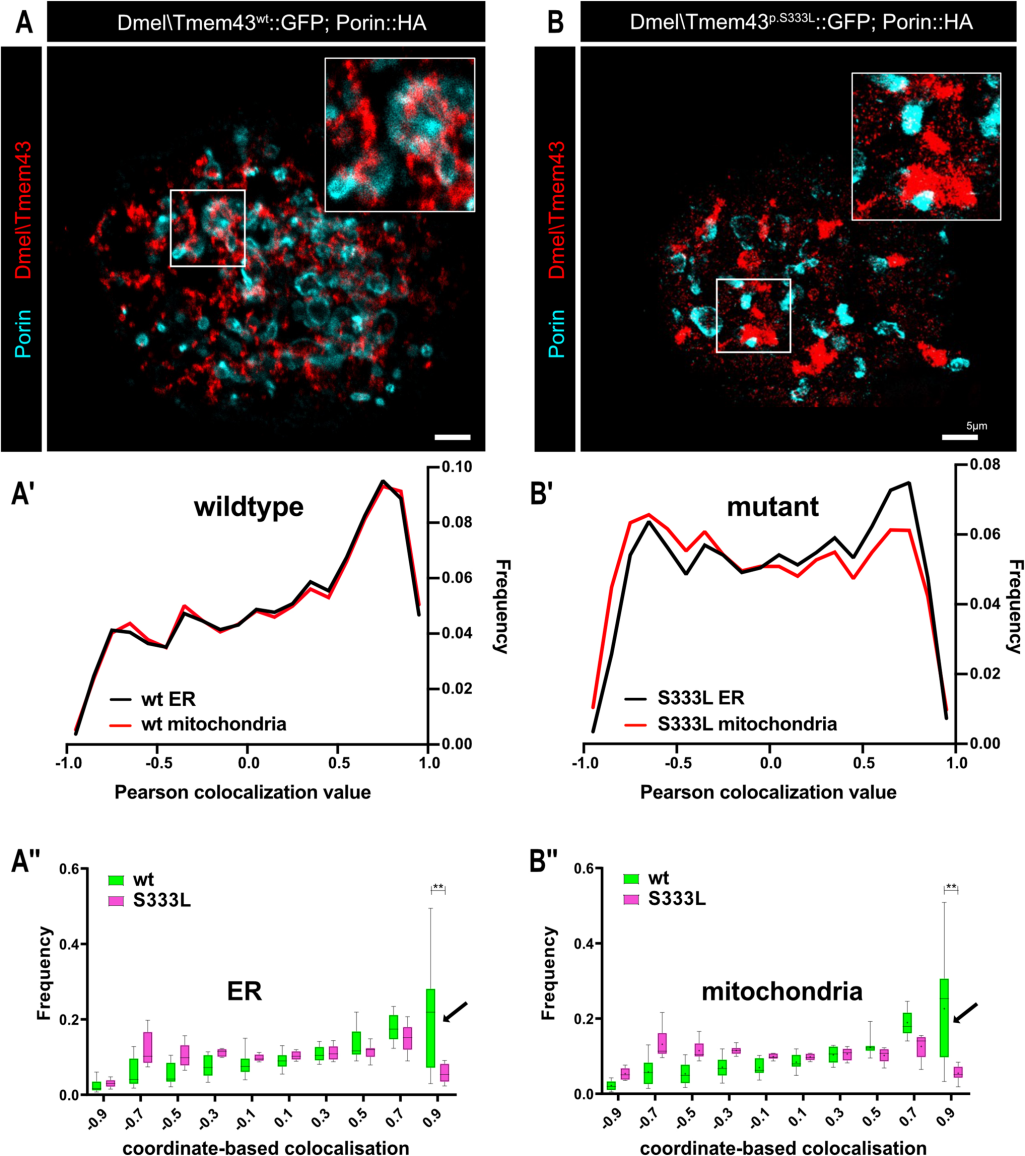
DNA-paint-based super-resolution imaging reveals impaired ERMCSs in Dmel\Tmem43p.S333L cells. *Sf*21 cells co-transfected with either Dmel\Tmem43wt::GFP and Porin::HA (**A**) or Dmel\Tmem43p.S333L::GFP and Porin::HA (**B**) are stained for GFP (green) and Porin (turquoise). (A´, B´) Quantitative analysis of the ER (black line) co-localisation coefficient or mitochondria (red line) Dmel\Tmem43 with Porin. (A´´, B´´) Quantification of the spatial distribution of either Dmel\Tmem43wt::GFP or Dmel\Tmem43p.S333L::GFP, relative to Porin::HA. A significantly reduced colocalisation is evident between Dmel\Tmem43p.S333L and Porin, relative to Dmel\Tmem43wt and Porin (arrows)

To assess whether Dmel\Tmem43wt colocalises with Porin at ERMCSs and whether the localisation pattern is impaired in the Dmel\Tmem43p.S333L mutant background, we applied DNA-PAINT-based super-resolution microscopy. *Sf*21 cells were cotransfected with either Dmel\Tmem43wt::GFP and Porin::HA (Fig. 3A) or Dmel\Tmem43p.S333L::GFP and Porin::HA (Fig. 3B) and stained for GFP and HA using DNA-paint suitable GFP-nanobodies and anti-HA antibodies. The image datasets were bioinformatically processed to quantify non-overlapping, partially overlapping, or fully overlapping signals for both proteins at the ER membrane, the mitochondrial membrane, or ERMCSs (Fig. 3A´, A´´, B´, B´´). The quantitative analysis revealed that about 0.5% of all Dmel\Tmem43^wt^ signals in the ER membrane were in direct proximity to mitochondrial Porin signals, presumably representing ERMCSs. If the same analysis was applied to cells transfected with the mutant Dmel\Tmem43p.S333L, a significantly reduced colocalisation between TMEM43 and Porin was observed, indicating a reduced number of ERMCSs in the mutant (Fig. 3A´, A´´, B´, B´´). Considering the critical roles that ERMCSs play in many biological processes, ranging from lipid synthesis and catabolism to calcium signalling and facilitation of cellular respiration, we analysed whether the apparent reduction in ERMCSs also affected the histology of the muscle cells.

### Colocalization between Dmel\Tmem43 and mito-ER tether constructs is impaired in mutant muscle tissue

To further confirm localization of Dmel\Tmem43 at the ERMCS, we utilised a previously established mitochondrial-ER tether construct [19],Csordas et al., 2006; [21], expressed under the control of *mef2.* We co-stained muscle tissue of 3rd instar larvae expressing either wildtype or mutant Dmel\Tmem43::HA constructs and the UAS-mito-ER tether to assess colocalization.

**Fig. 4:**
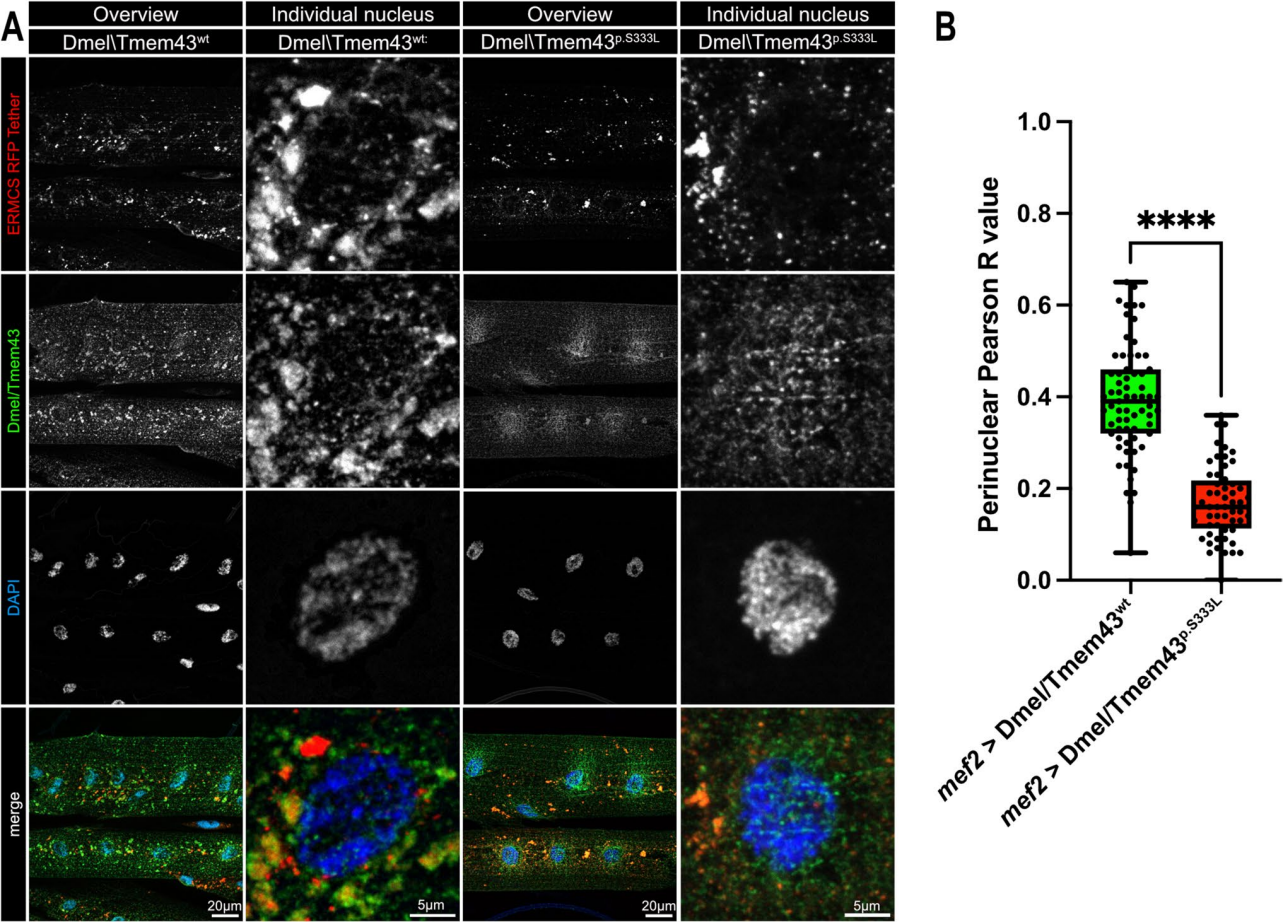
(**A**) confocal images of 3rd instar larval muscle tissue expressing either Dmel\Tmem43wt::HA (left) or Dmel\Tmem43p.S333L::HA (right), along with the ERMCS tether (UAS-mito-ER). All constructs are driven by *mef2-*Gal4. Overviews (1st and 3rd lane) and nucleus-specific images (2nd and 4th lane) are shown for both constructs. Dmel\Tmem43wt::HA constructs show strong colocalization with the tether, whereas mutant p.S333L::HA constructs exhibit a more diffuse pattern with a reduced overlap at ERMCS. (**B**) Quantification of the colocalization between wild-type or mutant Dmel\Tmem43::HA signals and UAS-mito-ER signal using Pearson's correlation coefficient. Significantly reduced colocalisation is evident for the mutant protein (unpaired t-test, p**** < 0.0001). Each point represents an individual nucleus

In Dmel\Tmem43wt::HA; UAS-mito-ER constructs, we observed a strong and punctate co-distribution of the signals around the nuclei. Interestingly, the mutant p.S333L variant exhibited a substantially different localization pattern. Corresponding colocalization values were significantly reduced in the mutants, suggesting impaired proximity or mislocalization of Dmel\Tmem43p.S333L::HA (Fig. 4).

### Dmel\Tmem43p.S333L affects mitochondrial structure and density in muscle cells

**Fig. 5:**
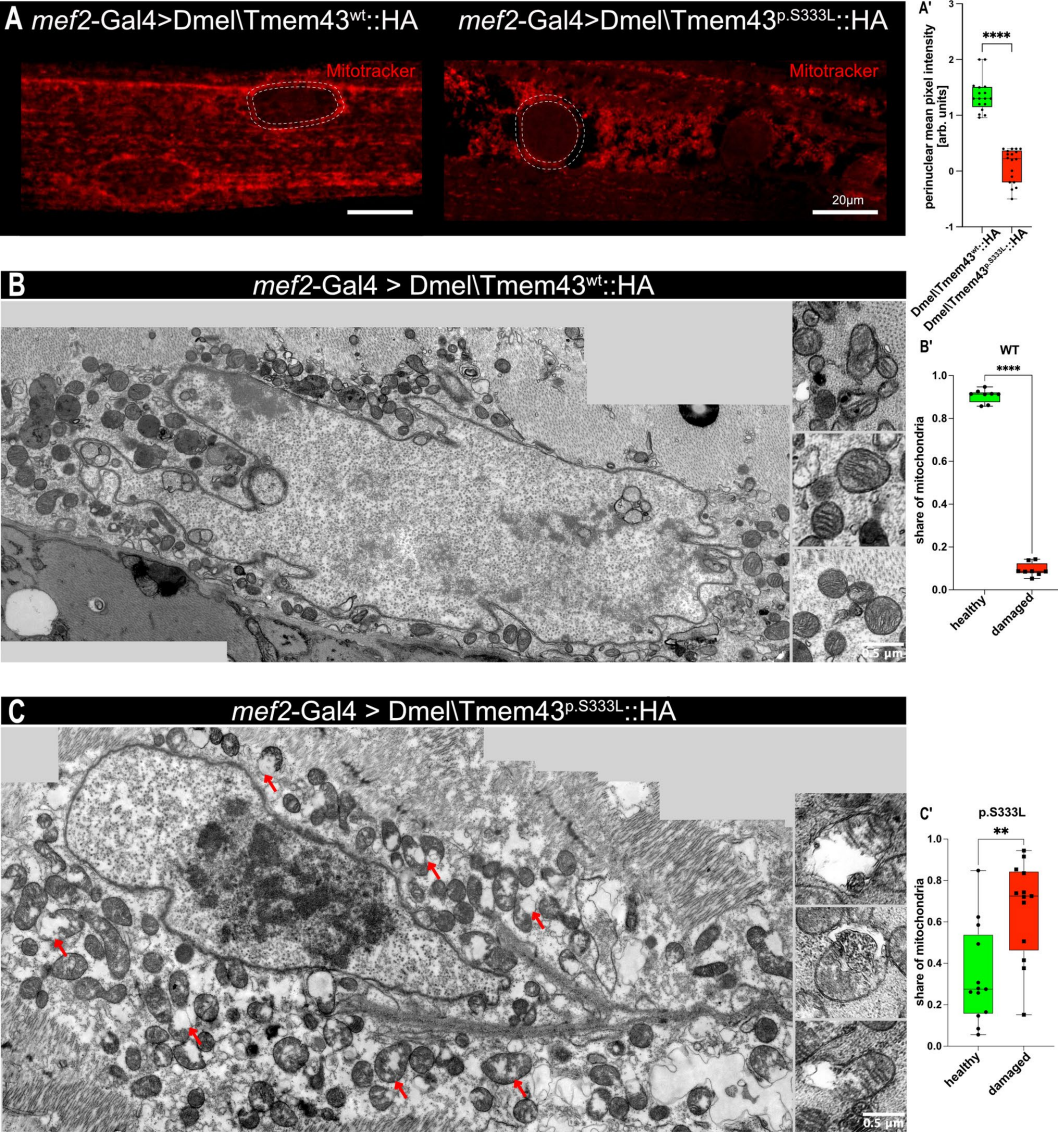
Expression of Dmel\Tmem43p.S333L induces mitochondrial damage in muscle cells. (**A**) Somatic muscles of transgenic 3rd instar larvae, expressing wild-type or p.S333L Dmel\Tmem43, were stained with Mitotracker to visualise functional mitochondria. (A´) The relative amount of perinuclear mitochondria in the respective genetic backgrounds was measured in a 10 µm ROI around the cell nuclei (dashed circles in A). The mean intensity of the individual mitotracker signals is depicted. Perinuclear mitochondria exhibit a significantly reduced abundance in the Dmel\Tmem43p.S333L mutants (unpaired t-test, p**** < 0.0001). (**B**) Representative transmission-electron micrograph of a somatic muscle from 3rd instar larvae expressing Dmel\Tmem43wt. Three mitochondria, which we consider ultrastructurally normal, are shown (insets). (B´) Quantification of „healthy“ and „damaged“ mitochondria reveals a significantly increased amount of healthy mitochondria in flies expressing Dmel\Tmem43wt (unpaired t-test, p**** < 0.0001). Approximately 90% of the mitochondria are classified as „healthy “. (**C**) Representative transmission-electron micrograph presenting a somatic muscle from 3rd instar larvae, expressing Dmel\Tmem43p.S333L. Three mitochondria, which we consider ultrastructurally abnormal, are exemplarily shown (insets). (C´) Quantification of „healthy“ and „damaged“ mitochondria reveals a significantly increased amount of damaged mitochondria in flies expressing Dmel\Tmem43p.S333L (unpaired t-test, p** < 0.01). Approximately 30% of the mitochondria are classified as „healthy“, while the remaining 70% exhibit different manifestations of damage, including swelling, cristae degradation, and bursting

We analysed the number and cellular distribution of mitochondria in transgenic flies expressing either Dmel\Tmem43wt::HA or the Dmel\Tmem43p.S333L::HA mutant protein via Mitotracker staining. For the mutant, considerably reduced numbers of perinuclear mitochondria were observed relative to the control (Fig. 5A). For quantification, we applied mean intensity measurements in the perinuclear mitochondria population. We found that the number of perinuclear mitochondria was significantly reduced in the mutant compared to the wild-type (Fig. 5A´).

Thus, we conclude that the expression of mutant Dmel\Tmem43p.S333L::HA disrupts the ordered arrangement of mitochondria in myocytes and may cause mitochondrial degeneration, resulting in reduced numbers of perinuclear mitochondria.

**Fig. 6:**
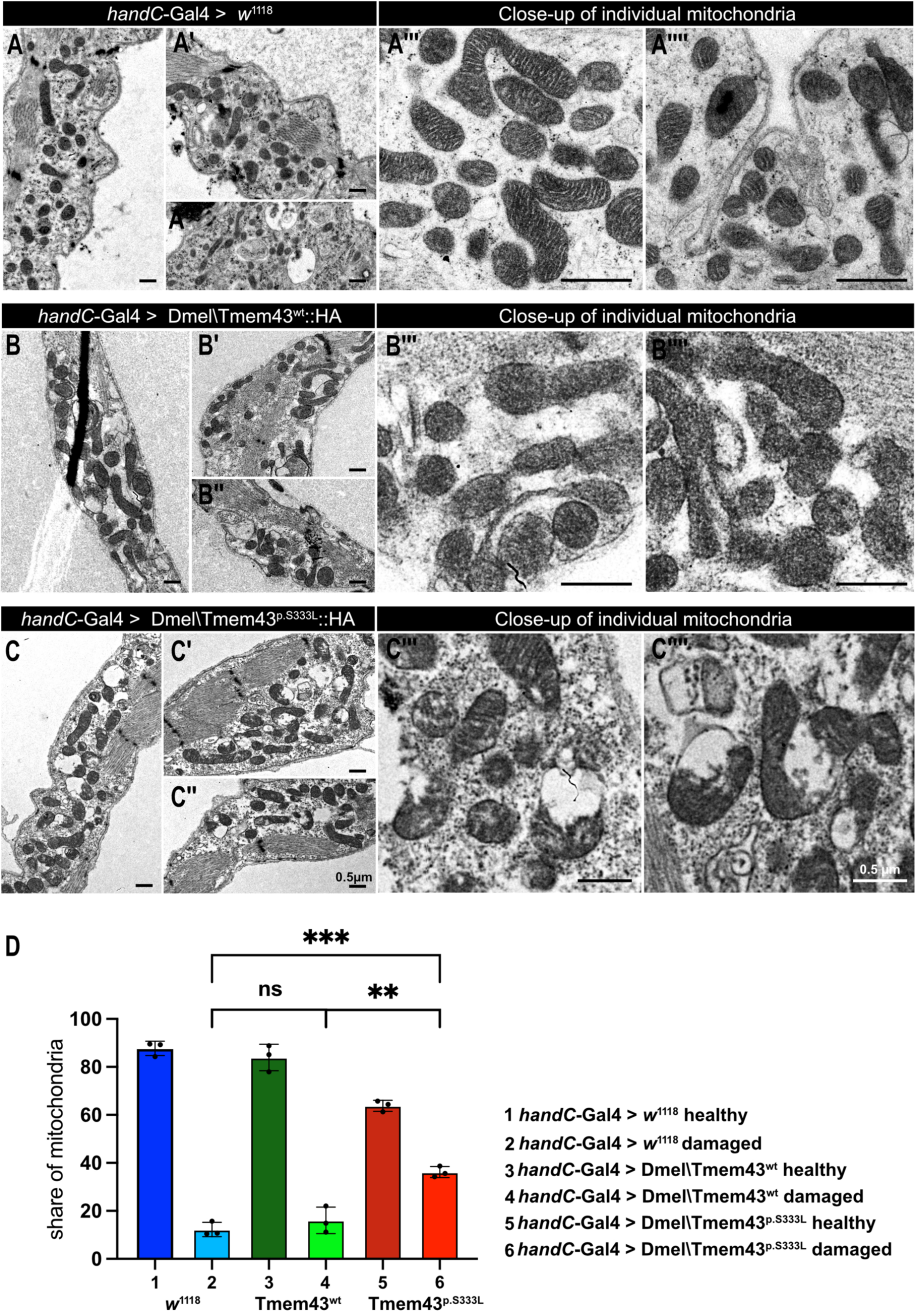
(**A**) Representative transmission-electron microscopy (TEM) images of a heart tube from control 3rd instar larvae (*w*^1118^ x *handC*-Gal4*)*. (A ‘, A ‘‘, A ‘‘‘, A ‘‘‘‘) Different orientations and close-ups of individual mitochondria are shown (insets). (**B**) Representative TEM images of a heart tube from transgenic 3rd instar larvae, expressing Dmel\Tmem43^wt^::HA driven by *handC-*Gal4. (B ‘, B ‘‘, B ‘‘‘, B ‘‘‘‘) Different orientations and close-ups of individual mitochondria are shown (insets). (**C**) Representative TEM images of a heart tube from transgenic 3rd instar larvae, expressing Dmel\Tmem43^p.S333L^::HA driven by *handC-*Gal4. (C ‘, C ‘‘, C ‘‘‘, C ‘‘‘‘) Different orientations and close-ups of individual mitochondria are shown (insets). (**D**) Quantification of damaged mitochondria for the different genotypes reveals significantly increased amounts of damaged mitochondria in mutant heart cells (unpaired t-test, p*** < 0.001; p** < 0.01, ns = not significant). Overexpression of the wild-type construct results in 84% healthy and 16% damaged mitochondria. For mutants, approximately 64% of the mitochondria are classified as „healthy“, while the remaining 36% exhibit different manifestations of damage, including swelling, cristae degradation, and bursting

To corroborate the light microscopic observations, we performed TEM ultrastructural analyses of either *mef2-*Gal4 or *handC*-Gal4 driven Dmel\Tmem43wt::HA or Dmel\Tmem43p.S333L::HA (Figs. 5B and C and 6), with *w*1118 serving as a control. In the mutant, a zone largely devoid of mitochondria was evident around the nuclei of muscle cells. For the remaining mitochondria, we observed mitochondrial swelling, outer membrane rupture, intramitochondrial vacuolisation, and degradation of cristae structures. Corresponding impairments were only rarely detected in control mitochondria. We used histological criteria to classify mitochondria as either healthy or damaged. This approach does not provide information about function but serves to statistically document the mitochondria's appearance. By quantifying and comparing the percentage of mitochondria that appear damaged with those that look intact, based on the phenotypic characteristics described above, we found that 66% of the mitochondria in the perinuclear area were damaged in mutant flies. In contrast, only 9.4% of the mitochondria in control flies were classified as damaged.

To confirm occurrence of the described effects also in cardiomyocytes, we utilised heart-specific *handC*-Gal4 as a driver. We observed a similar phenotype as already seen for the *mef2* constructs (Fig. 6). Again, mitochondria in the heart cells were defective and exhibited severe structural abnormalities. However, compared to the *mef2*-Gal4 crossings, the *handC*-Gal4 specific effects were less pronounced, revealing 16% damaged mitochondria in wildtype flies and 36% in flies carrying the p.S333L mutation.

### Human TMEM43p.S358L patients show mitochondrial damage in cardiomyocytes

**Fig. 7:**
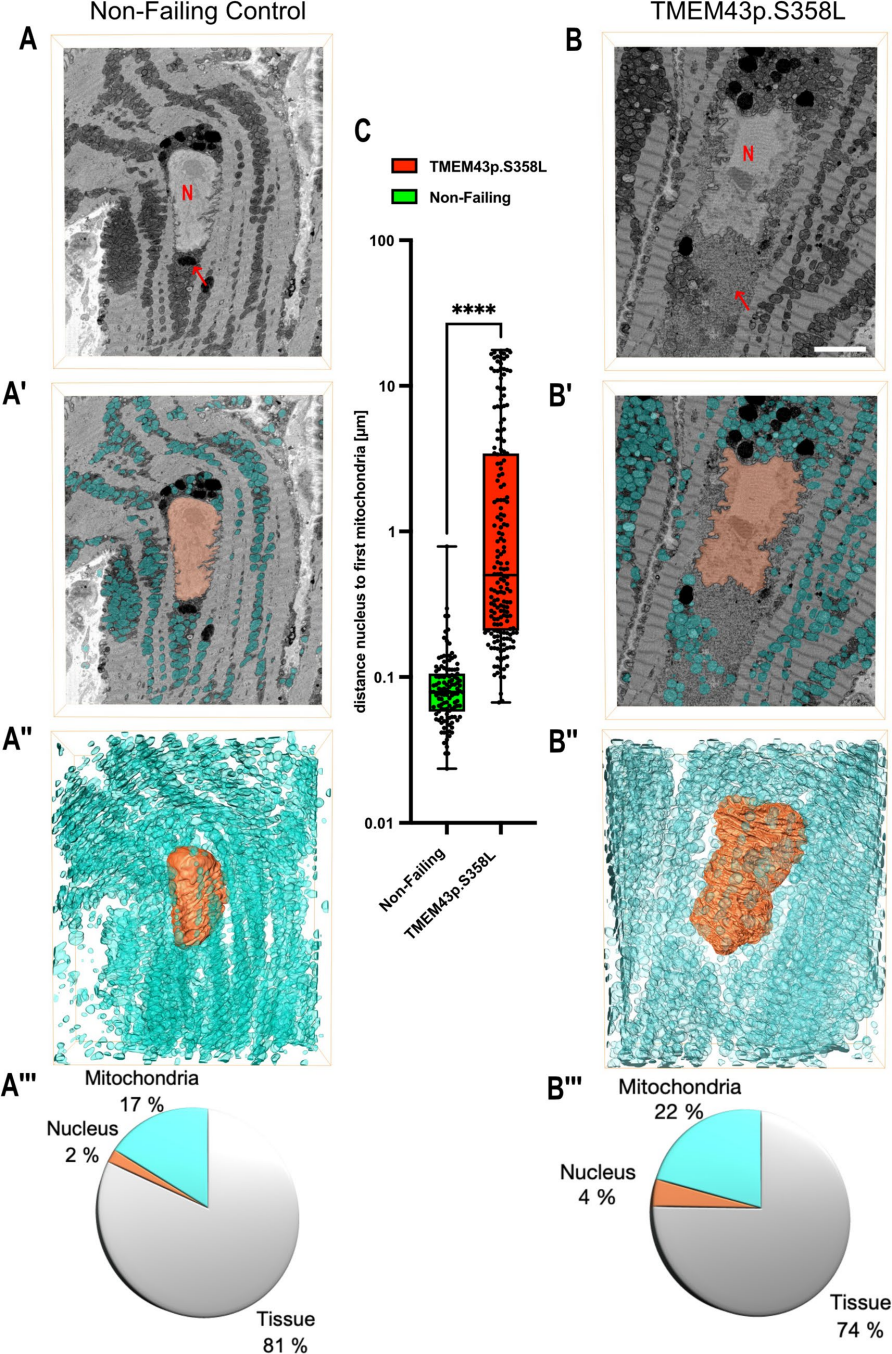
Ultrastructural analysis of a TMEM43p.S358L carrier patient´s heart reveals mitochondrial damage. (**A**, **B**) Three-dimensional volumetric transmission electron microscopy was applied to analyse the ultrastructure of human cardiomyocytes isolated from the right ventricular myocardium. (A) Tissue from a rejected donor heart (control). The image shows a single slice of the complete image stack. The nucleus appears as a light-grey area (N). The zone with perinuclear mitochondria is indicated (red arrow). (**B**) Tissue from a TMEM43p.S358L carrier. The nucleus appears as a light-grey area (N). The mitochondria-devoid area around the nucleus is indicated (red arrow). (**C**) Quantification of mitochondria in the perinuclear area. A significant reduction of perinuclear mitochondria is evident in cardiomyocytes of the p.S358L heart (unpaired t-test, p**** < 0.0001). (A´, A´´ and A´´´) Control. (B´, B´´ and B´´´) TMEM43p.S358L carrier. (A´, B´) Single slice from the 3D-reconstruction of the volumetric TEM image data set, processed with the KI-based Empanada algorithm for mitochondrial segmentation. The reconstruction allows us to visualise and measure the number, size, distribution and accumulation of mitochondria (blue) in the control and the TMEM43p.S358L heart tissue. The cell nucleus is coloured orange. (A´´, B´´) 3D reconstruction of representative nuclei (orange). The nucleus in the TMEM43p.S358L cardiomyocyte appears enlarged compared to the control nucleus. (A´´´, B´´´) Quantification of the volumes occupied by the nucleus and the sum of all mitochondria. The total volume of the measured area was set to 100%. Nucleus volume and total mitochondrial volume are increased in TMEM43p.S358L cardiomyocytes (3 cells were analysed per genotype), indicating mitochondrial swelling

Recently, a 21-year-old TMEM43p.S358L male carrier was admitted to the Heart and Diabetes Center NRW, Bad Oeynhausen, Germany, for heart transplantation. Myocardial samples from the right ventricle of the explanted heart were processed as previously described [1] and subjected to proteomics, lipidomics and NMR-based metabolomic analyses. The results will be presented in detail in a separate study by our team [17]. For the present analysis, tissue samples of the explanted heart were processed for volumetric 3D-electron microscopy (for technical details, see method section). Rejected non-failing donor hearts (NF) served as a control. For serial block-face SEM analysis, 500 µm^3^ samples were generated to produce about 500 individual slices (50*50*25 µm^3^). Single images were aligned and assembled into 3D images, allowing sample analysis at different section levels, angles, and resolutions (Supplementary Fig. 5). We found histological features in the patient´s heart largely consistent with the data obtained from the *Drosophila* muscle tissue expressing the p.S333L mutant (Fig. 5C). As a remarkable coincidence, the perinuclear mitochondria were degenerated and had a much lower density than in the NF-control heart. By quantifying the distance from the outer mitochondrial membrane facing the nucleus to the nuclear lamina as a measure of the mitochondria-free area around the nuclei, we confirmed significant differences in the respective mitochondrial perinuclear populations (Fig. 7A, B and C). While the mean distance of perinuclear mitochondria to their corresponding nucleus was 0.1 µm (0,09445 µm) in the NF-control, in the TMEM43p.S358L carrier tissue, it extended to approximately 3 µm (3,018 µm) distance (Fig. 7C). This increased distance in TMEM43p.S358L tissue was observed in the longitudinal and the transversal SBF datasets.

Further measurements using 3D reconstructions revealed an increased mitochondrial volume in TMEM43p.S358L cardiomyocytes compared to NF heart cells (Fig. 7A´´´, B´´´). Swollen mitochondria occur due to mitochondrial malfunction and often precede organelle degeneration. These observations suggest that the expression of Dmel\Tmem43p.S333L and TMEM43p.S358L similarly affect mitochondrial structure and function in flies and humans. To further substantiate this indication, we analysed the mitochondrial membrane potential, a critical parameter of mitochondrial function, in cultured cells expressing either the wild-type or the mutated form of Dmel\Tmem43.

### Dmel\Tmem43p.S333L causes the collapse of the mitochondrial membrane potential

**Fig. 8:**
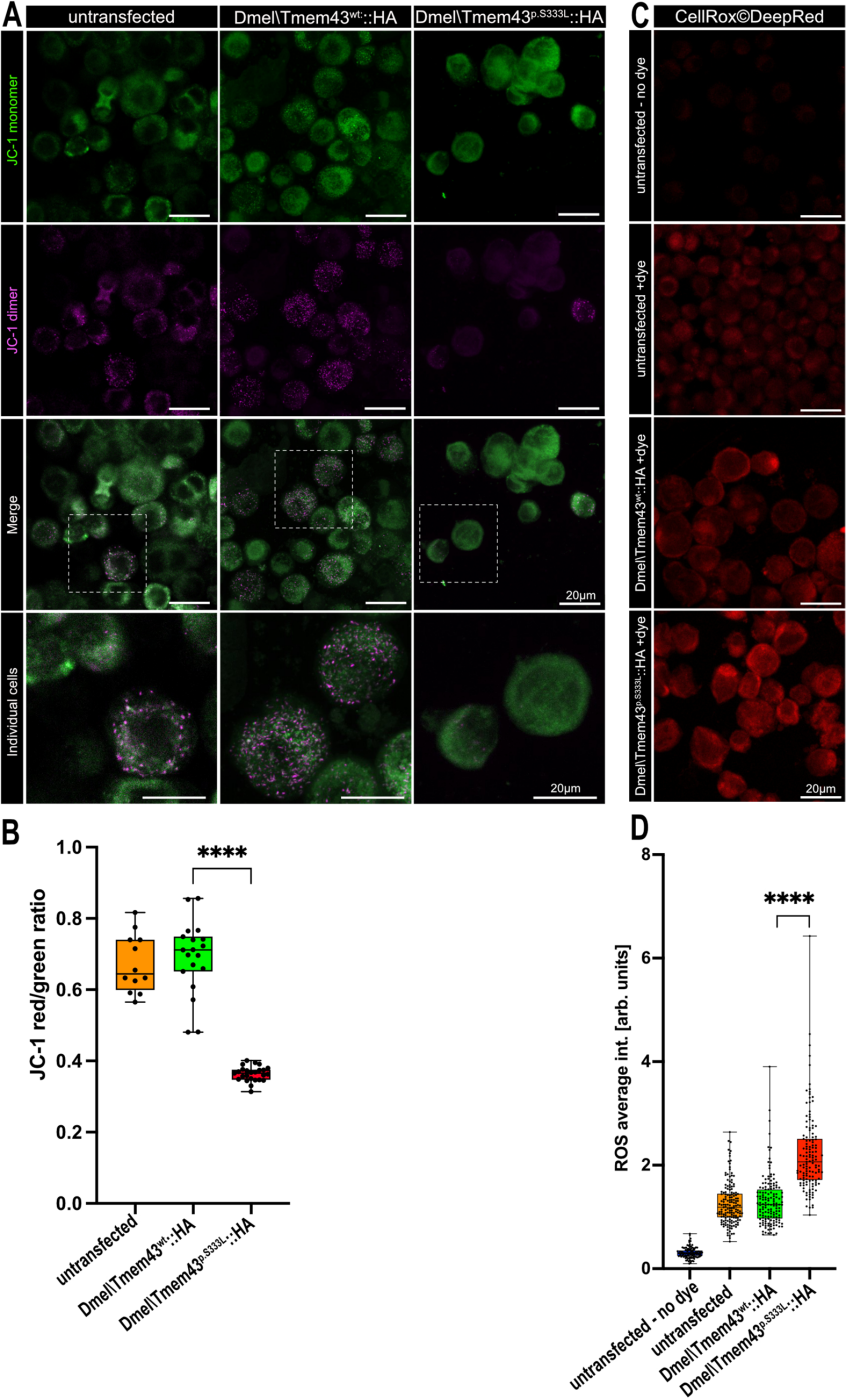
Abnormal mitochondrial membrane potential and ROS accumulation indicate an impaired mitochondrial function in Dmel\Tmem43p.S333L expressing cells. (**A**) *Sf*21 cells untransfected (left column) or transfected with either Dmel\Tmem43wt::HA (middle column) or Dmel\Tmem43p.S333L::HA (right column) were stained with JC-1 dye. The lowest panel shows the corresponding images at higher magnification (indicated by dashed squares). In cells transfected with Dmel\Tmem43p.S333L::HA, the red signal emitted by JC-1 dimers is mainly absent, indicating a breakdown of the mitochondrial membrane potential. (**B**) Quantification of the green/red fluorescence ratio in the respective cells. Presence of Dmel\Tmem43p.S333L::HA results in significantly decreased mitochondrial membrane potential (unpaired t-test, p**** < 0.0001). (**C**) *Sf*21 cells transfected with either Dmel\Tmem43wt::HA or Dmel\Tmem43p.S333L::HA were stained with CellRox DeepRed dye to estimate intracellular ROS concentrations. Untransfected cells without the addition of dye (background noise) and untransfected cells with the dye added were used as individual controls. (**D**) Quantification of the ROS signal intensities. The average fluorescence intensity is significantly increased in *Sf*21 cells expressing Dmel\Tmem43p.S333L::HA, relative to cells expressing Dmel\Tmem43wt (unpaired t-test, p**** < 0.0001). This indicates significant ROS accumulation in the cells expressing the mutant construct

To substantiate our hypothesis that the mitochondria in Dmel\Tmem43p.S333L mutants are dysfunctional and, therefore, prone to degeneration, we labelled *Sf*21 cells transfected with either Dmel\Tmem43wt or Dmel\Tmem43p.S333L with JC-1. The JC-1 dye accumulates in healthy mitochondria in a membrane potential-dependent manner. The accumulation can be visualised by aggregation of the dye, which is accompanied by a shift in emission from green (~ 529 nm) for the monomeric form to red (~ 590 nm) for the aggregates. Thus, a decrease in the red/green fluorescence intensity ratio indicates mitochondrial depolarisation, predicting mitochondrial dysfunction. Quantification revealed that *Sf*21-cells expressing the mutant Dmel\Tmem43p.S333L protein contained a significantly higher number of depolarised, presumably largely inactive mitochondria than cells expressing wild-type Dmel\Tmem43 (Fig. 8A and B). Therefore, we conclude that the mutant Dmel\Tmem43p.S333L affects oxidative phosphorylation and, thus, ATP production.

### Reactive oxygen species accumulate in cells expressing Dmel\Tmem43p.S333L

Depolarisation of mitochondria indicates a reduced respiratory chain efficiency and impaired function of the organelle. Numerous studies in animal models and humans have shown that mitochondrial dysfunction is often associated with increased reactive oxygen species (ROS) production [24]. Of note, elevated ROS levels can, directly and indirectly, promote the opening of the mitochondrial permeability transition pore (PTP). The PTP is a channel with high conductance. Its opening leads to a loss of Δψm and uncoupling of oxidative phosphorylation, resulting in mitochondrial swelling and outer membrane rupture (Korge et al., 2017). We also observed these phenotypes in cells expressing Dmel\Tmem43p.S333L (Fig. 5C). Consequently, we analysed corresponding *Sf*21 insect cells for the accumulation of ROS. Untransfected cells and cells transfected with wild-type Dmel\Tmem43 were used as individual controls. Interestingly, *Sf*21 cells expressing the mutant Tmem43 protein were characterised by significantly increased ROS levels relative to both controls (Fig. 8C and D).

**Fig. 9:**
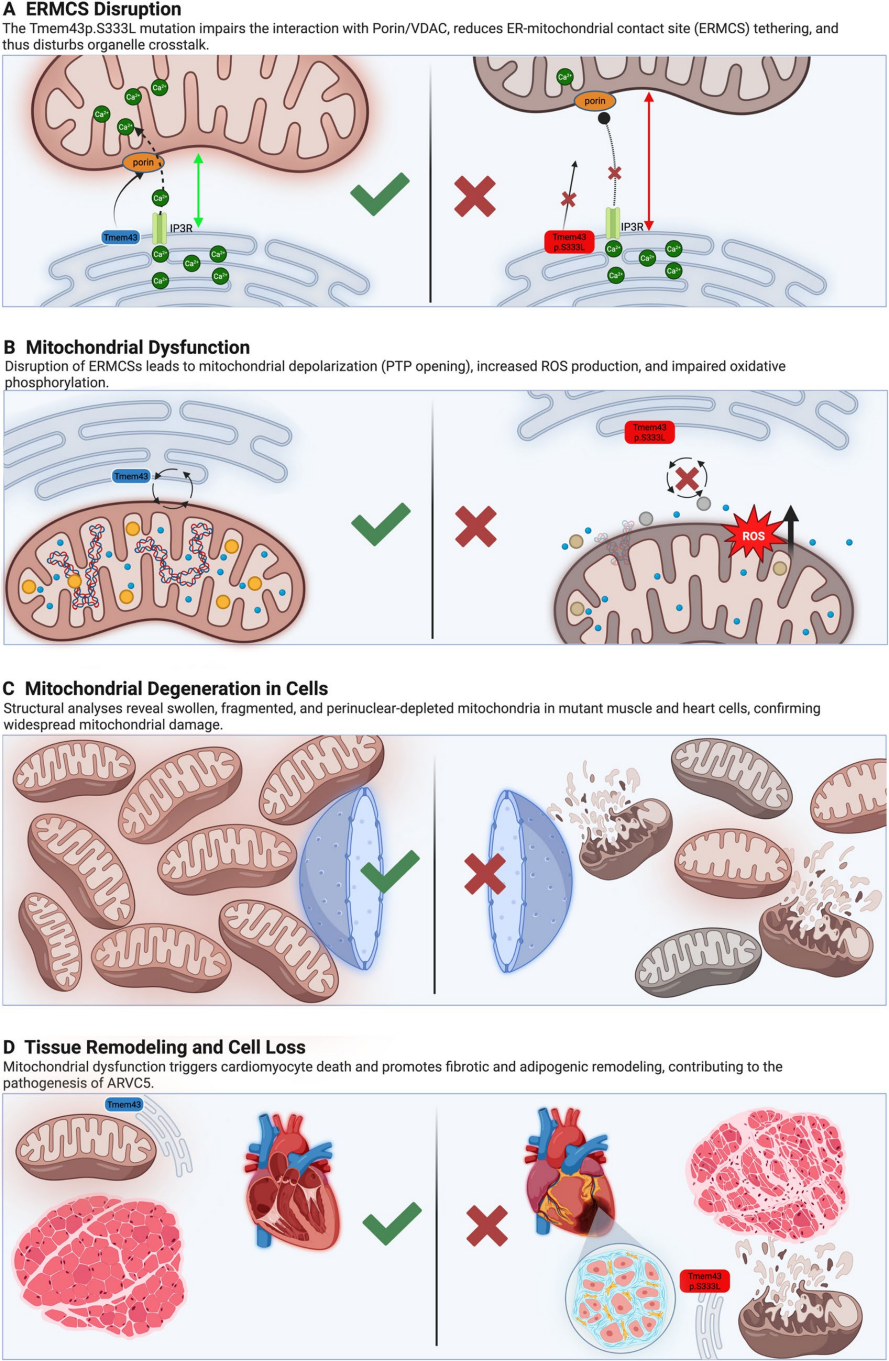
Schematic representation of the detrimental effects of Dmel\Tmem43p.S333L. The p.S333L mutation in Dmel\Tmem43 causes ERMCS disruption (**A**), mitochondrial dysfunction (**B**), mitochondrial degeneration (**C**), tissue remodeling, and cell loss (**D**). This eventually leads to heart failure and sudden cardiac death in humans with a pathogenesis typical of ARVC type 5. We propose that the mutant Tmem43 protein disrupts Porin-dependent organellar communication, mediated by the ER mitochondrial contact sites, leading to impaired mitochondrial membrane potential and function. Over time, mitochondria are progressively and irreversibly damaged. This leads to insufficient ATP production and loss of cardiomyocyte contractility. As a result, the muscle tissue progressively degenerates and undergoes fibro-fatty replacement. Created in BioRender. Jürgens, K. (2025) https://BioRender.com/1l6oxus

These data further substantiate that the variant Dmel\Tmem43p.S333L (TMEM43p.S358L) predominantly impairs proper mitochondrial function and oxidative phosphorylation rates. The resulting undersupply of ATP likely leads to the progressive death of myocytes and, ultimately, to heart failure, which is an uncommon disease mechanism in ARVC (Fig. 9).

## Discussion

### TMEM43p.S358L-induced cardiomyopathy is induced by mitochondrial dysfunction

ARVC-5 is a detrimental disease caused by the missense mutation TMEM43p.S358L. We applied a combination of cultured cells, transgenic *Drosophila*, and cardiac tissue from an explanted human heart carrying the corresponding mutation to better understand the molecular basis of TMEM43-related ARVC-5. Our data prove that the relevant missense mutation in TMEM43 induces mitochondrial dysfunction. Both *Sf*21 cells and *Drosophila* muscle cells of the corresponding genotype accumulate mitochondria with numerous disease-relevant phenotypes, including membrane-potential breakdown, increased ROS levels, perinuclear mislocalisation, cristae degradation, and mitochondrial lysis (Figs. 5, 6 and 8). Significantly, the mitochondrial phenotypes observed in the *Drosophila* model correspond to the histological phenotypes evident in a TMEM43p.S358L patient´s heart. Volumetric transmission electron microscopy of myocardial tissue from the explanted right ventricle revealed mitochondrial swelling, perinuclear mitochondrial degradation, and the remains of membranous structures around the nuclei (Fig. 7). These data indicate that the expression of Dmel\Tmem43p.S333L in flies and TMEM43p.S358L in humans affects mitochondrial structure and function. It has long been known that specific cardiomyopathies are caused by mitochondrial dysfunction, e.g., by mutations in respiratory chain proteins that affect the energy balance of the cardiomyocytes and, thus, muscle cell function. Of note, recent studies showed that genetically and physiologically induced malfunctions of VDAC proteins impair mitochondrial calcium homeostasis, among other things [25–28]. This, in turn, leads to a disturbed energy balance of the mitochondria, which ultimately initiates mitophagy or mitochondrial degeneration. In this scenario, cardiomyopathies occur due to an undersupply of energy in cardiomyocytes.

### Mitochondrial dysfunction in Dmel\Tmem43p.S333L and TMEM43p.S358L mutants may be caused by impaired ER-mitochondrial contact sites (ERMCSs)

Growing evidence shows that impaired ER/SR-mitochondria tethering, mediated by specific contact sites (ERMCSs) in heart cells, is critical for heart failure [29]. Recent studies showed that the so-called mitochondria-associated ER membrane (MAM) comprises up to 20% of the outer mitochondrial membrane. In these areas, the ER and the mitochondria are separated by a gap of only 10–25 nm, with a connection between the organelles being established by distinct protein complexes [30]. The resulting contact zones are crucial for exchanging numerous molecules such as lipids, metabolites, and calcium [31]. Significantly, calcium enhances oxidative phosphorylation via stimulating calcium-sensitive matrix dehydrogenases of the TCA cycle. Given the high energy demand of cardiomyocytes and the need to balance the rate of mitochondrial ATP production with utilisation over a wide range of activity, this regulation of ATP production is an essential factor for proper heart function [32]. Our super-resolution DNA-PAINT-based microscopy studies detected wild-type and mutant Dmel\Tmem43 in distinct punctual microcompartments along the ER membrane (Fig. 3A and B). Co-staining with Porin/VDAC, the anion-exchange channel located in the OMM, revealed that the Dmel\Tmem43-positive compartments are close to Porin-positive OMM spots (Fig. 3A and B). Of note, the distance between the Dmel\Tmem43 and the Porin signals was in the range of 20–30 nm. Moreover, in line with recent data indicating interaction between TMEM43 and VDAC1 at the protein level [33], our proximity biotinylation and pull-down analyses also validated the interaction between Dmel\Tmem43 and Porin (Figs. 1 and 2). Thus, the observed overlapping signals likely represent ER/SR-mitochondrial contact sites. Whether these sites represent a new, unidentified class or belong to a known group of ERMCSs remains to be determined. Intriguingly, quantitative analysis of the DNA-paint datasets revealed that the number of Dmel\Tmem43p.S333L-Porin contact sites was considerably lower than that of corresponding Dmel\Tmem43wt::Porin sites. This altered pattern may be caused by the conformational change that occurs in the third TM-domain of Dmel\Tmem43p.S333L, relative to Dmel\Tmem43wt [1] and the presumably altered protein–protein interactions with other surrounding factors within the SR membrane or the OMM. This possibility is strongly supported by the fact that wild-type Dmel\Tmem43 efficiently interacts with Porin, while the p.S333L mutant lost its ability to interact with the channel (Fig. 2E, E´, F, F´).

To provide further evidence that Dmel\Tmem43 localizes to ERMCS in vivo, we analysed the colocalization of wildtype and mutant constructs with UAS-mito-ER, a well-characterized mitochondrial-ER tether [19],Csordas et al., 2006; [21]. We found a clear colocalization of wild-type Dmel\Tmem43::HA with the tether. This suggests an important function of Dmel\Tmem43 at the ERMCS. Interestingly, the mutant construct failed to resemble the wild-type expression pattern. Relative to the wild-type protein, presence of the p.S333L mutation lead to reduced colocalization between the protein and the tether, suggesting an impaired localization of Dmel\Tmem43p.S333L, with reduced abundance at ERMCS. This is consistent with our previous biochemical and DNA-Paint data that indicated close proximity and interaction of wild-type Dmel\Tmem43 and mitochondrial Porin, which was significantly affected in mutant Dmel\Tmem43. Dmel\Tmem43p.S333L consistently exhibited a reduced localization to the ERMCS.

Considering also our TEM and JC1 data that indicated impaired mitochondrial structure and function as a result of p.S333L introduction, we conclude that relative to the wild-type protein, mutant Tmem43 establishes or maintains a reduced number of ER/SR-mitochondrial contact sites per cell. This likely results in disturbed organellar communication and metabolite/ion exchange. As a result, membrane potential and ROS production within mitochondria are severely impaired, eventually inducing mitochondrial death and organelle degradation. The consequential shortage of ATP would then induce cardiac cell death and the observed replacement of muscle cells by fibrotic and fat tissue. Our observation of reduced mitochondrial abundance in the perinuclear area of Tmem43 mutants in both *Drosophila* and human myocardium further supports this scenario and indicates a high transferability of the data obtained in the *Drosophila* model. Of note, recent studies showed that the perinuclear mitochondria mainly regulate nucleoplasmic calcium signalling and are associated with heart failure [34].

Our results introduce impaired oxidative phosphorylation and mitochondrial function as major physiological defects in an ARVC-5-relevant TMEM43 *Drosophila* model. Reduced stability or abundance of ERMCS and a resulting misregulation of organellar communication likely represent the mechanistic bases of the phenotypes. Our finding that highly similar morphological defects were also present in the myocardium of a TMEM43p.S358L carrier suggests a common molecular basis for the detrimental effects of the mutation in flies and humans.

## Methods

### Note

The *Drosophila* orthologue of the human *TMEM43* gene was initially annotated in the Flybase database as CG8111 (CG = CeleraGenomics). However, we named the gene throughout this manuscript “Dmel\Tmem43” for easy reading. All constructs and transgenic *Drosophila* stocks were listed in our laboratory files with the original CG8111 nomenclature. Therefore, we use “CG8111” instead of “Dmel\Tmem43” in this Methods section.

### Fly stocks

The following fly stocks were previously described or generated during this study. Cloning details are available upon request.

UAS-CG8111^WT^ and UAS-CG8111^p.S333L^ [1]

UAS-CG8111^WT^::HA and UAS-CG8111^p.S333L^::HA [1].

UAS-CG8111^WT^-TurboID and UAS-CG8111^p.S333L^-TurboID (generated),

CG8111^p.S3333L^ = Dmel\Tmem43^p.S333L^ CRISPR-Cas9 induced mutant (generated),

*mef2*-GAL4 (BL #27390) and *tub*-GAL4 (BL #5138).

w[1118]; P{w[+ mC] = UAS-mito.RFP.ER.B}H/TM6B, Tb[1] (BL #95120).

### Mitotracker stainings, immunostainings in larvae and Sf21 cells

*Sf*21-cells (derived from *Spodoptera frugiperda*) were cultured in Insect-Xpress medium (Lonza, Basel) at 28 °C. Protein expression and immunostaining in *Sf*21 cells were performed as described previously [35, 36]. The working concentration of MitoTracker was 1:250 for muscle tissue from 3rd instar larvae. Stainings were performed according to the manufacturer's instructions (Thermo Fisher, New Hampshire, USA). Immunohistochemistry in larvae was performed as previously described [37].

### Antibodies

The primary antibodies used were rabbit anti-CG8111 (1:100, [1]), mouse or rabbit anti-HA (1:1000, DSHB), and anti-GFP (1:200, Abcam). Secondary antibodies used were goat anti-mouse-Cy3 (1:200, Dianova), goat anti-mouse AF488 (1:200, Dianova) and anti-rabbit Cy3 (1:200, Dianova). StreptavidinAF488 (1:200, ThermoFisher) was used to visualise biotinylated proteins in tissue preparations.

### Confocal microscopy and processing of LSM images

If not otherwise noted, all confocal LSM images were captured with a Zeiss LSM800 laser scanning microscope. Image processing was done with Fiji and Affinity Photo (Serif).

### Pull-down and Western blots

The pull-down experiments and Western blots were carried out as described previously [1, 38]. Nitrocellulose membranes were imaged with an Odyssey Clx Imager (LI-COR, Lincoln,NE, USA), and quantification was performed using Empiria Studio 1.1 Software (LI-COR).

### TurboID and streptavidin staining

*Drosophila* 3rd instar larvae overexpressing Dmel\Tmem43wt::TurboID or Dmel\Tmem43p.S333L::TurboID in a muscle-specific manner were used for proximity labelling. Larvae were grouped into cohorts of 10 animals, and five cohorts per genotype were independently analysed to identify possible interaction partners of Dmel\Tmem43. Animals were collected and frozen at -70 °C until usage.

To homogenise the larvae, they were frozen in liquid nitrogen (LN2), ground to powder, and again frozen in LN2. 300 μl lysis buffer + 1 × protease inhibitor (Promega, Madison, WI, USA) was added to the sample and vortexed until the powder was resolved. Afterwards, samples were centrifuged for 15 min at 10,000 rpm at 4 °C. The supernatant was transferred to a new reaction cup, and 50 μl of streptavidin microbeads (Miltenyi Biotech, Bergisch Gladbach, Germany) were added. The mixture was incubated for 30 min on ice. During the incubation, μ-columns (Miltenyi Biotech) were prepared for further use. Therefore, the μ Columns were pre-incubated with 200 μl lysis buffer. After 30 min, samples were run through the μ-columns twice. Subsequently, μ-columns were washed 2 × with 200 μl lysis buffer to remove unbound proteins. Ensuing, 50 μl reduction solution was added, and μ-columns were incubated for five minutes at room temperature, followed by another incubation of 30 min at 38 °C. Afterwards, μ-columns were washed 2 × with 200 μl wash buffer. Then, 50 μl alkylation solution was added, and μ-columns were incubated for 15 min at room temperature in darkness. Subsequently, μ-columns were washed 2 × with 100 μl wash buffer. To remove the wash buffer from the μ-columns, 25 μl digestion buffer and 25 μl digestion buffer + 0.01 μg/μl trypsin was added. Digestion took place overnight at 30 °C. The next day, 25 μl digestion buffer was added to the μ-columns and incubated for five minutes. Finally, the eluate, containing cleaved peptides, was collected in 1.5 ml reaction cups and further analysed by mass spectrometry.

To ensure that the generated lines carrying the fused construct express the GOI consistently, 3rd instar larvae were pinned on a Sylgard® plate. With the help of a binocular, larvae were pinned down with minutiae (Austerlitz) at the anterior and posterior ends. To make sure that the larvae do not dry out, a drop of 1X PBS was added. After this, the ventral side of the larvae was cut open. First, a perpendicular cut to the anterior–posterior axis was performed at the posterior end. After that, the larvae were cut from pin to pin to open the ventral side. The cuticle was fixed with two minutiae on each side before dissecting the larvae for the desired purpose of the experiment.

After dissection, larvae were fixed in 4% methanol-free formaldehyde on an orbital shaker for one hour. Larvae were washed for 3 × 10 min with BBT (1 × PBS (1,4 M NaCl, 27 mM KCl, 18 mM KH_2_PO_4_, pH = 7,2), 0.1% Tween-20, 0.1% BSA) to remove formaldehyde. The following steps were performed in a 1.5ml reaction cup. The samples were incubated in permeabilisation buffer (1 × PBS, 1% Triton X-100) for one hour before washing the sample with BBT again (3 × 10 min). To prevent unspecific binding of the dye, samples were treated with saturation buffer (1 × PBS, 1% BSA, 0,1% Tween-20) for one hour. Streptavidin AlexaFluor488 dye, diluted in saturation buffer, was added and subsequently incubated in the dark at RT 60 min. Larvae were washed for 3 × 10 min with BBT to remove unbound remains of the dye conjugate. Eventually, larvae were mounted on microscopy slides in Fluoromount-G mounting medium containing DAPI (ThermoFisher).

### Mass spectrometry

Dried peptides were resuspended in 10 µl LC-Load, resulting in a protein concentration of 100 ng/ul. 1ul was used to perform reversed-phase chromatography on a Thermo Ultimate 3000 RSLC nano system connected to a TimsTOF HT mass spectrometer (Bruker Corporation, Bremen) through a Captive Spray Ion source. Peptides were separated on a Aurora Gen3 C18 column (25 cm x 75um × 1.6um) with CSI emitter (Ionoptics, Australia) at temperature of 40 °C and eluted from the column via a linear gradient of acetonitrile from 10–35% in 0.1% formic acid for 44 min at a constant flow rate of 300 nl/min following a 7 min increase to 50%, and finally, 4 min to reach 85% buffer B.

Eluted peptides were directly electro sprayed into the mass spectrometer at a electrospray voltage of 1.5 kV and 3 l/min Dry Gas.

The MS settings of the timsTOF were adjusted to positive Ion polarity with a MS range from 100 to 1700 m/z. The scan mode was set to DIA-PASEF.

The ion mobility was ramped from 0.7 Vs/cm2 to 1.5 in 100 ms. The accumulation time was also set to 100 ms. 10 PASEF ramps per cycle resulted in a duty cycle time of 1.17 s. The target intensity was adjusted to 14000, the intensity threshold to 1200. The dynamic exclusion time was set to 0.4 min to avoid repeated scanning of the precursor ions, their charge state was limited from 0 to 5.

The data were loaded to PeaksOnline Version 1.9 and analysed with the PeaksOnline workflow (DeNovo and DB Search) with Precursor mass error tolerance of 15 ppm, Fragment Mass error tolerance of 0.5 Da, CSS Error tolerance of 0.05 and a Missed Cleavage of 2. As modification Carbamidomethylation(C) (fix) and Oxidation(M) (variable) was chosen.

As protein database the *Drosophila-*specific database (UP000000803, www.uniprot.org/proteomes/UP000000803) was used. The results were filtered for peptides with a FDR of 1%, for proteins by the -10lgP of 20. Classification as a possible interaction partner required *p* < 0.05, with quantification based on at least two individual protein-specific peptides.

### Three-dimensional exchange DNA-PAINT imaging

Viral-transfected *Sf*21 cells expressing His-tagged Porin and either wild-type Dmel\Tmem43 or mutated Dmel\Tmem43 tagged with mEGFP were fixed with 4% PFA for 30 min at RT. Afterwards, the cells were washed with PBS, permeabilised with 2% BSA and 0,1% Triton in PBS, and labelled with an anti-His tag antibody (Sigma (H6908)) diluted 1:100 overnight at 4 °C. Afterward, cells were washed three times with PBS and labelled overnight at 4 °C with an anti-GFP nanobody (MASSIVE-TAG-X2-FAST anti-GFP; Massive Photonics GmbH, Gräfelfing, Germany) and an anti-rabbit nanobody (MASSIVE-sdAB-FAST 1-PLEX; Massive Photonics GmbH, Gräfelfing, Germany), both diluted 1:100 (50 nM) in PBS containing 3% BSA and 0.1% Triton-X-100. Before imaging, the cells were washed three times with PBS and incubated with a 1:10 dilution of 90 nm gold particles (CG-90–20; Cytodiagnostics) for 5 min at room temperature, followed by another wash with PBS. These nanoparticles functioned as fiducial markers during image acquisition and for channel alignment. After that, cells were washed three times with PBS before an imaging buffer (500 mM NaCl, 0.1 mM EDTA, 0.05% Tween in PBS) containing approximately 125 pM ImagerF2-Cy3b was added. This imaging DNA strand is complementary to the DNA strand attached to the anti-rabbit nanobody, which allows the identification of transfected cells via the mEGFP signal for Dmel\Tmem43 and the blinking behaviour of Porin. Cells were imaged using an inverted microscope frame (Olympus IX-81) equipped with a motorised quad-line TIR illumination condenser (cellTIRF-4-Line, Olympus) adjusted in HILO mode and a motorised xy-stage (Märzhäuser Scan IM 120 × 80). Three-dimensional single-molecule localisation was achieved by astigmatic imaging using a cylindrical lens (Olympus) implemented directly in front of the filter wheel. While the mEGFP signal was excited with a 488 nm diode-pumped solid-state laser (max. power 150 mW, Olympus), imager strands containing Cy3b were excited with a 561 nm diode-pumped solid-state laser (max. power 150 mW, Olympus), passing through a 100 × oil immersion objective (UAPON 100 × TIRF, NA 1.49, Olympus). Excitation intensities for imager strands were typically adjusted to 20–30 W/cm^2^. Fluorescence emission was filtered by bandpass filters (BrightLine HC 525/25 for mEGFP and BrightLine HC 600/37 for Cy3b, Semrock) before being recorded with a sCMOS camera (ORCAFlash 4.0 V3, Hamamatsu). CellSens 2.2 (Olympus) was used as the acquisition software to record 60,000–80,000 frames, with an exposure time of 50 ms per frame and a 2 × 2 pixel binning that resulted in a pixel size of 130 nm. The sample focus plane was stabilised during acquisition via a hardware autofocus system (IX2-ZDC2, Olympus). The temperature was kept stable at 25 °C with a large incubator chamber (TempController 2000–2, Pecon) while the sample was humidified via a small stage-top incubator (CO_2_-Controller 2000, Pecon) to prevent buffer evaporation during long-term imaging. After imaging of the ImagerF2-Cy3b, cells were washed with PBS until no ImagerF2 binding could be detected. Then, imaging buffer containing approximately 125 pM ImagerF3-Cy3b was added, complementary to the DNA strand attached to the anti-GFP nanobody. Once again, 60,000–80,000 frames were acquired under the same imaging conditions as before. Three-dimensional localisation required axial calibration of astigmatic PSFs by acquiring z-stacks of immobilised fluorescent TetraSpeck™ microspheres with a diameter of 100 nm (Invitrogen, T7279). Stacks were recorded in an imaging buffer with a step size of 10 nm using a piezo z-stage (NanoScanZ, NZ100, Prior Scientific).

### DNA-PAINT data analysis

Raw data sets were processed with the “Picasso” software package [39] (https://github.com/jungmannlab/picasso). First, the calibration z-stack of TetraSpeck™ beads was analysed using “Picasso Localize.” The box size was set to 13 to identify single beads and the Min. Net. Gradient was adjusted to filter out weak signals from unwanted background localisations. Photon conversion parameters were set as follows: EM Gain: 1, Baseline: 400, Sensitivity: 0.46, Quantum Efficiency: 0.80, and Pixel Size: 130 nm. A calibration file was generated with the “Calibrate 3D” function of “Picasso Localize.” This file, along with the same photon conversion parameters, was used to process raw sample files. The Min. Net Gradient was adjusted to remove nonspecifically bound imager strands and filter for localisations with the highest signal intensities. Single-molecule localisations were fitted using a least-squares Gaussian fit. For 3D localisation, the magnification factor was set to 1.0. Each processed dataset representing an image area was opened with “Picasso Render,” and a drift correction was first conducted using cross-correlation, followed by a correction with gold nanoparticles as fiducials. After that, both channels were aligned via cross-correlation and the fiducials. The localisations of these datasets were then exported for the ImageJ plugin ThunderSTORM for further image processing and visualisation [40].

### Coordinate-based colocalization (CBC) analysis

For quantitative colocalisation analysis of the three-dimensional exchange DNA-PAINT imaging, we utilised a Coordinate-Based Colocalization (CBC) analysis implemented in the ThunderSTORM plugin for ImageJ [41]. This approach employs the spatial coordinate data of each localisation, rather than their intensity, to evaluate colocalisation between fluorescently labelled proteins. In CBC analysis, each protein localisation is assigned a CBC value ranging from − 1 to + 1, quantifying the spatial correlation between the two analysed proteins. A CBC value of − 1 indicates an anti-correlated distribution, suggesting a low probability of colocalisation; values close to 0 indicate a lack of correlation; and values approaching + 1 signify a high probability of colocalisation, reflecting a perfectly correlated distribution. For our analysis, we assessed 12 cells expressing wild-type Dmel\Tmem43 and nine cells expressing mutated (p.S333L) Dmel\Tmem43.

### mito-ER tether analysis

Mitochondrial ER tether constructs (mito-ER) were combined with a *mef2-*Gal4 driver line via crossover. Resulting flies were crossed with the Dmel\Tmem43 constructs to observe the tethering complexes via RFP fluorescence in vivo and to analyse colocalization of the tether with Dmel\Tmem43 constructs. Dissection and immunostaining were performed as described above. The antibodies used were anti-HA (1:200, mouse, Abcam) and anti-mouse AF488 (1:200, Dianova GmbH). DAPI was used as a counterstain and applied with the secondary antibody at 1:100.

After LSM imaging, z-stacks of tissues were generated, 300 × 300px around every nucleus were cut out, and Coloc2 analysis within Fiji was performed. The resulting Pearson values were collected, statistically evaluated, and plotted in Prism 10 (GraphPad, Boston, MA, USA).

### Transmission electron microscopy

After TEM images of the relevant perinuclear, or heart cell environments were acquired, panoramic images were generated. These panoramic images were then analysed by visually assigning the mitochondria into two categories. Healthy mitochondria required an intact outer mitochondrial membrane and a visible cristae structure, whereas defective mitochondria were categorised as missing the outer membrane, bursting into the cytosol, or showing signs of degraded cristae structure. Percentages of these categories were calculated for each imaged environment and then further processed using GraphPad Prism10.

### Sample preparation for volumetric 3D electron microscopy

Tissue biopsies were initially cryo-fixated in liquid nitrogen and subsequently shipped to the Integrated Bioimaging Facility (IBiOs) in Osnabrück, Germany. To mitigate ultrastructural damage induced by ice crystals, samples were slowly warmed to -20 °C, then thawed on ice in a cold primary fixative (2.5% glutaraldehyde (EMS, #16120) in 0.1 M cacodylate buffer, pH 7.4 (Science Services, #E11652)) with gentle agitation for 24 h. The recovered tissue samples were then cut into smaller pieces, approximately 1 mm^3^, to facilitate adequate penetration and diffusion of the subsequent mega-metal staining required for serial block-face analysis.

An adapted version of the original Ellisman protocol [42, 43] ensured enhanced contrast and stable image acquisition. In brief, samples were washed in buffer and then post-fixed in 2% osmium tetroxide (Science Services, #19140) and 1.5% potassium hexacyanoferrate (Science Services, #E11652) in the buffer for 2 h on ice. All subsequent steps were performed at room temperature (RT), with thorough rinsing in double-distilled water between each step on a shaker. After the initial osmication, samples were incubated for 20 min in aqueous thiocarbohydrazide (Sigma Aldrich, #223220), followed by a 30-min treatment with 2% OsO_4_, and then exposed to 1% tannic acid (MERCK, #403040) in water. The samples were incubated overnight in 1% uranyl acetate (EMS, #22400) at 4 °C.

The following day, samples were brought to 50 °C, washed in warm water, and then incubated in freshly prepared Walton’s lead aspartate for 30 min at 60 °C (composed of lead(II) nitrate (Carl-Roth, #10099–74-8), L-aspartate (Serva, #14180.02), and KOH (Merck, #109112)). Dehydration was achieved using aqueous ethanol in increasing concentrations (20%, 50%, 70%, 90%), followed by two washes in pure ethanol on ice, succeeded by replacement with first ice-cold and then RT anhydrous acetone (VWR Chemicals, #83683.23), for 10 min each. Samples were then infiltrated with graded mixtures of acetone and Epon 812 (Sigma Aldrich, #45359-1EA-F; hard formula) (25%, 50%, 75%) for 2 h each, followed by overnight incubation in pure resin. Subsequently, samples were transferred into fresh Epon for an additional 24 h and then cured at 60 °C for 48 h.

Once fully polymerised, tissue blocks were trimmed into frustum shapes of approximately 300 µm^3^ and glued onto aluminium stubs using epoxy-based conductive adhesive (Microtonano, #AG29D). Samples were then examined for cellular integrity and orientation of sarcomeres via toluidine blue staining. Those deemed suitable for further analysis were coated with 20 nm of gold and mounted within the 3View2XP chamber (Gatan, Pleasanton, CA, USA), fitted into a JSM 7200 F (JEOL Ltd., Tokyo, Japan).

Regions of interest were identified by creating large-scan overviews before acquiring a higher-resolution image series. Stable imaging was achieved under an accelerating voltage of 3.1 kV in high vacuum mode, with a 30 nm condenser aperture and 450 V of positive stage biasing. Imaging parameters were configured to a voxel size of either 5 × 5x50 or 3 × 3x25 nm, respectively, with a dwell time of 3 µs. Overall, eight image stacks with varying dimensions, based on the trajectories of individual sarcomeres, were acquired. A representative image stack consisted of 2000 2D sections with an image size of 12000 × 10000 pixels. Before image analysis, a focus on complete volumes of cardiomyocytes was set, approaching them either longitudinally or vertically. To minimise imaging time, the field of view was adjusted according to the development of the cardiomyocytes within the tissue block. Stage movement and image acquisition were controlled using Gatan Digital Micrograph (Version 3.32.2403.0).

### Image analysis and segmentation

The individual images were first aligned, converted to 8-bit, filtered, and cropped using Microscopy Image Browser software (MIB, [44]). The final image stacks were exported, typically binned by factors of 2 or 4, to maintain computational efficiency.

To extract the ultrastructural context and generate accurate statistics, the acquired volumes were segmented either in Empanada [45] for mitochondria or in MIB for nuclei. The MitoNet-based Napari-Plugin Empanda allows for comprehensive segmentation of mitochondria while enabling fine-tuning of models based on specific datasets. Due to the frequent occurrence of dynamic fission and fusion events and the close arrangement of mitochondria within cardiomyocytes, accurate object splitting and avoiding over-segmentation presented challenges, even after models were refined. Therefore, for individual measurements, a manual revision of the dataset was performed. For the analysis of nuclei volumes and surface areas, a subvolume of 10 slices was manually segmented and then used as ground truth to train a 2D Deep Learning Model (Z2C + DLv3 ResNet50) (DeepMIB) with a patch size of 768 × 768x5 pixels over 100 epochs. Following the prediction, manual corrections were made to the dataset.

Final models were binned to isometric voxel sizes and utilised for statistical analysis within MIB.

### Nucleus to mitochondrial distance evaluation

The Z-Stacks obtained from the serial block-face (SBF) analysis were used to analyse the distance of the nuclear membrane to the first contact of the mitochondrial membrane to adumbrate the amount of undefined tissue that obtains the perinuclear space in TMEM43p.S358L patient tissue. A mid-nucleus position was chosen as a base layer in which the shortest distance to the first perinuclear mitochondria was measured. It is important to note that mitochondria inhabiting the surrounding muscle-specific tissue were excluded to highlight the effect in the perinuclear mitochondria population surrounding the nucleus at opposite sides. After both sides were measured, four more Z positions with a distance of Z = 20 were evaluated according to the same method. This was done for both the longitudinal and transversal datasets obtained during SBF imaging.

### Mitochondrial volume

To analyse the amount of mitochondrial volume inside of the respective samples, three types of 3D segmentation models were created using MIB: mitochondria, nuclei, and surrounding tissue, specific to the cell in question. Using the evaluation tools of MIB, volumes were calculated for all three of them to gain insight into the relationship between the different tissues.

### Mitotracker staining - perinuclear mitochondria evaluation

Two ROI's were generated per nucleus using Fiji. The first ROI aligned with the outer nuclear membrane. Once this ROI was added to the ROI manager (Fiji), the ROI was scaled by 1.15 × to create a second ROI. Mean intensity values of both ROIs were evaluated and then the nucelar ROI mean intensity was subtracted from the scaled-up ROI to represent the mean intensity of the area between the two ROIs. The resulting values reflect mitochondrial population around the nucleus.

### JC-1 mitochondrial membrane potential assay

*Sf*21-cells were plated on 6-well plates containing a coverslip. After 30 min, 100 µl of respective virus particles were added. The wells were incubated at 28 °C for 72 h before further treatment. The coverslips with the *Sf*21-cells were transferred into humidity chambers. Cells were washed with 100 µl of 1 × dilution buffer (Abcam) before incubating the cells with 5 µM of JC-1 (Abcam) in dilution buffer for 10 min at 37 °C. Before mounting in Fluoromount-G (ThermoFisher), cells were washed three times with 1 × dilution buffer. Images were acquired with an LSM800 (Zeiss) and evaluated with the ROI manager and measurement tools provided by the ImageJ Software.

### ROS levels

*Sf*21 cells were cultured in 6-well plates containing coverslips using Insect-Xpress medium (Lonza, Basel, Switzerland). Thirty minutes after plating, 100 µL of the corresponding virus particles were added. The cells were incubated at 28 °C for 72 h, after which the coverslips were transferred into humidity chambers. Then, the cells were incubated in 5 µM CellROX™ Deep Red (ThermoFisher) in Insect-Xpress medium at 28 °C for 30 min. To ensure proper dispersion, the dye was vortexed for 20 s. This step had to be repeated before every incubation with the dye. Cells were washed 3 times with 1 × PBS exchange, fixed with 3.7% formaldehyde for 15 min, and mounted in Fluoromount-G (ThermoFisher). Images were captured using an LSM800 (Zeiss). Processing of the images was performed with the ROI manager and measurement tools provided by ImageJ Software.

### Statistical analysis

For statistical analyses, Graph Pad Prism 10 was used. Datasets were analysed for normal distribution using the D’Agostino & Pearson test. If normally distributed, the one-way ANOVA test was used. Otherwise, the Kruskal–Wallis test was applied. If only two datasets were compared, an unpaired t-test was performed. All values of histograms are means ± SD. For all boxplots, the centre line of a plot indicates the median; the upper and lower bounds indicate the 75th and 25th percentiles, respectively; and the whiskers indicate the minimum and maximum. The proteomics data were statistically analysed and visualised with PEAKS Studio software. All experiments were performed at least in triplicates.

## Supplementary Information

Below is the link to the electronic supplementary material.

Supplementary file1 (PDF 6170 KB)

Supplementary file2 (XLSX 429 KB)

Supplementary file3 (MP4 13991 KB)

Supplementary file4 (MP4 11593 KB)

## Data Availability

The datasets generated during the current study are available from the corresponding author on request.
