# Supplementary material for: The ARVC-5-associated protein TMEM43 controls mitochondrial energy metabolism by stabilising ER-mitochondrial contact sites
Source: Cell Mol Life Sci. 2025 Nov 14;82(1):400. doi: 10.1007/s00018-025-05942-z (PMC12618784; doi:10.1007/s00018-025-05942-z)
Supplement: Supplementary file 1 — Supplementary file1 (PDF 6170 KB) [file 18_2025_5942_MOESM1_ESM.pdf]

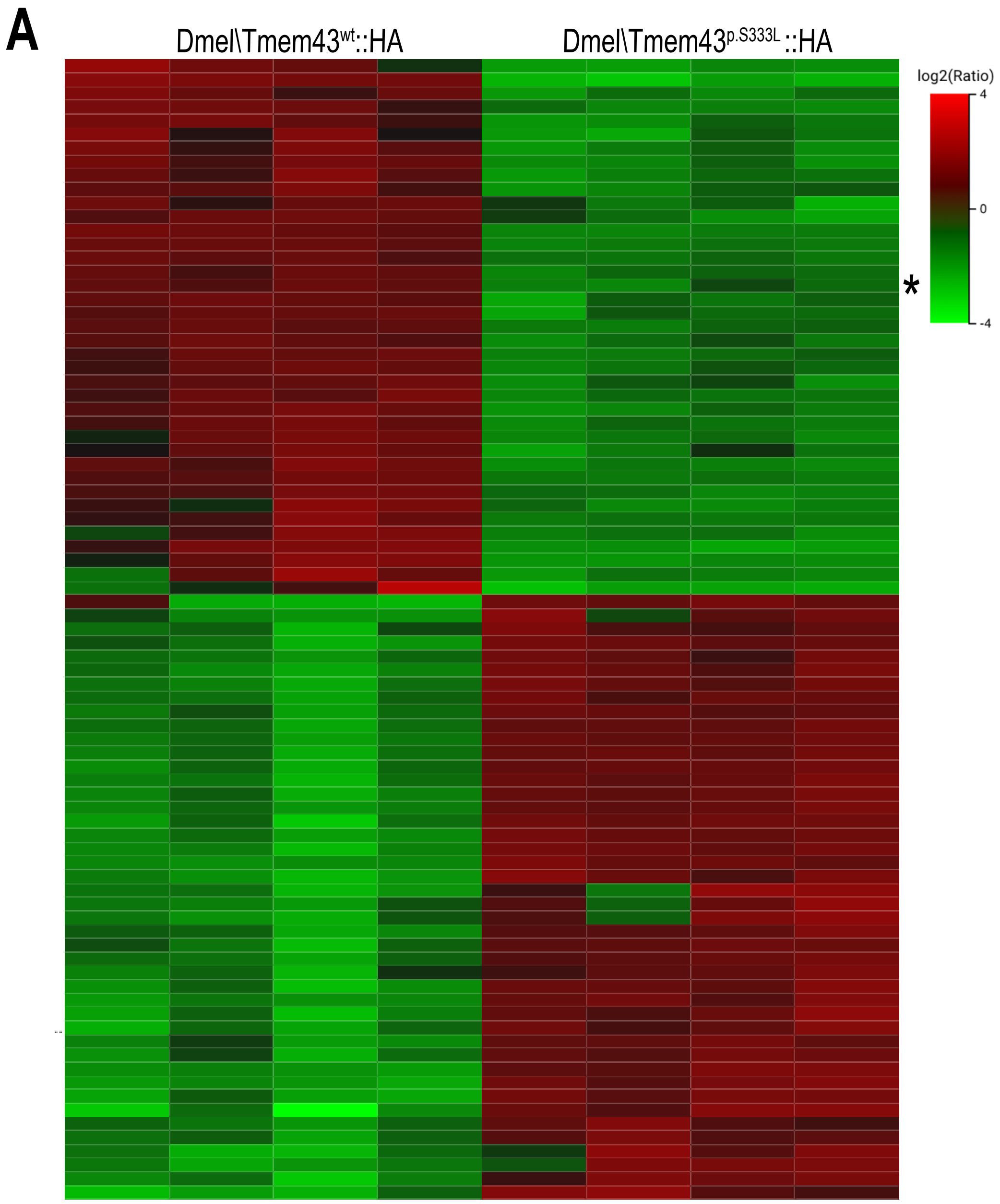

Supplementary Figure 1: Heatmap depicting the results of pull-down assays using Dmel\Tmem43wt::HA or Dmel\Tmem43p.S333L::HA as bait. Proteins were expressed in 3rd instar larval muscle tissue (*mef2*-Gal4). Porin significantly co-precipitates with Dmel\Tmem43wt::HA but not with Dmel\Tmem43p.S333L::HA (asterisk). Data are based on four individual biological replicates.

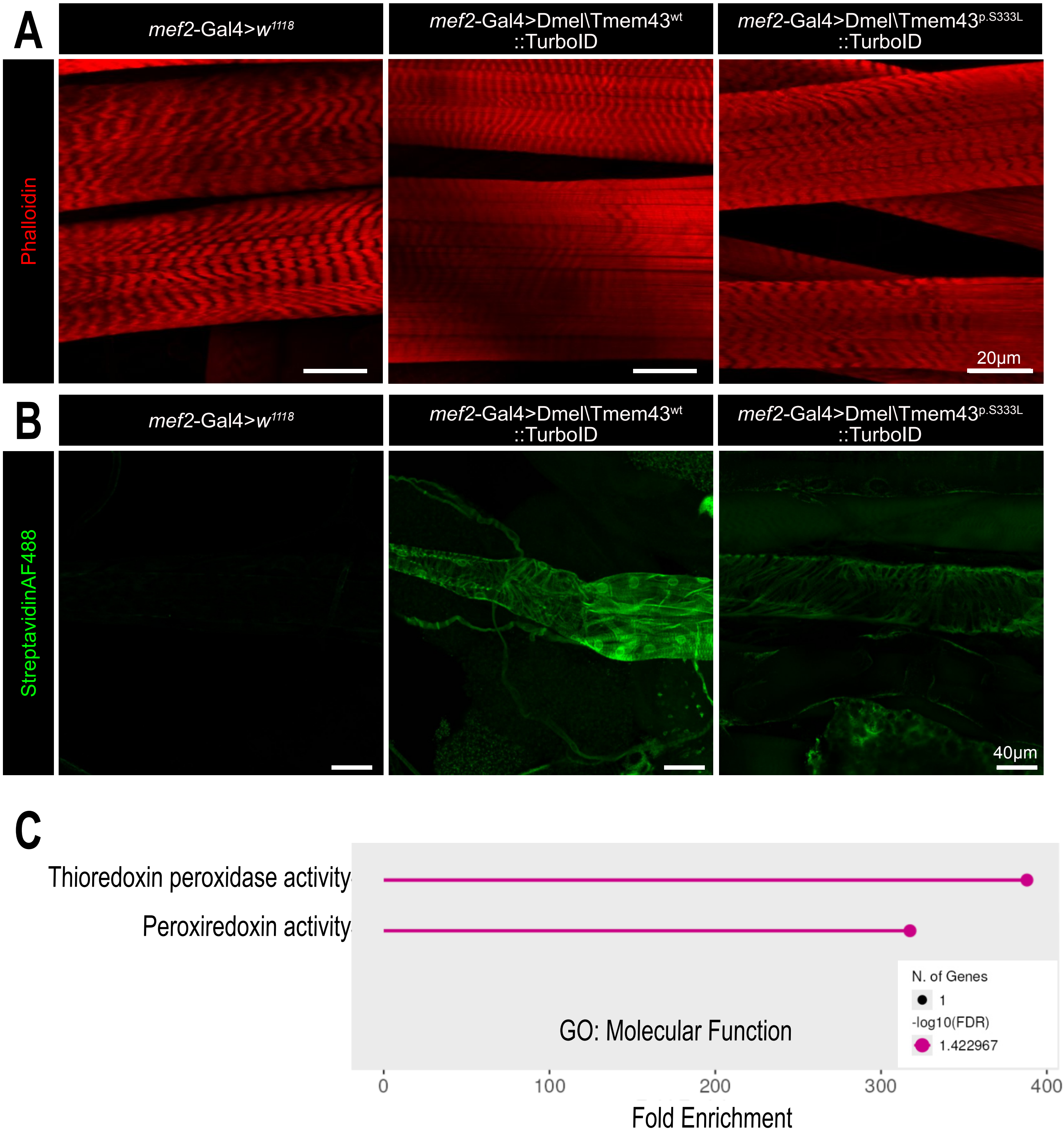

Supplementary Figure 2: (A) Phalloidin staining of 3rd instar larval body wall muscles expressing either wild-type Tmem43 (Dmel\Tmem43wt::TurboID) or mutated Tmem43 (Dmel\Tmem43p.S333L::TurboID). The driver line (*mef2-Gal4)* crossed to *w*1118 served as a control. (B) Streptavidin AlexaFluor488 staining of 3rd instar larval hearts expressing either wild-type Tmem43 (Dmel\Tmem43wt::TurboID) or mutated Tmem43 (Dmel\Tmem43p.S333L::TurboID). The driver line (*mef2-Gal4)* crossed to *w*1118 served as a control. (C) Gene Ontology (Molecular Function) analysis of the proteins enriched in Dmel\Tmem43p.S333L::TurboID compared to Dmel\Tmem43wt::TurboID.

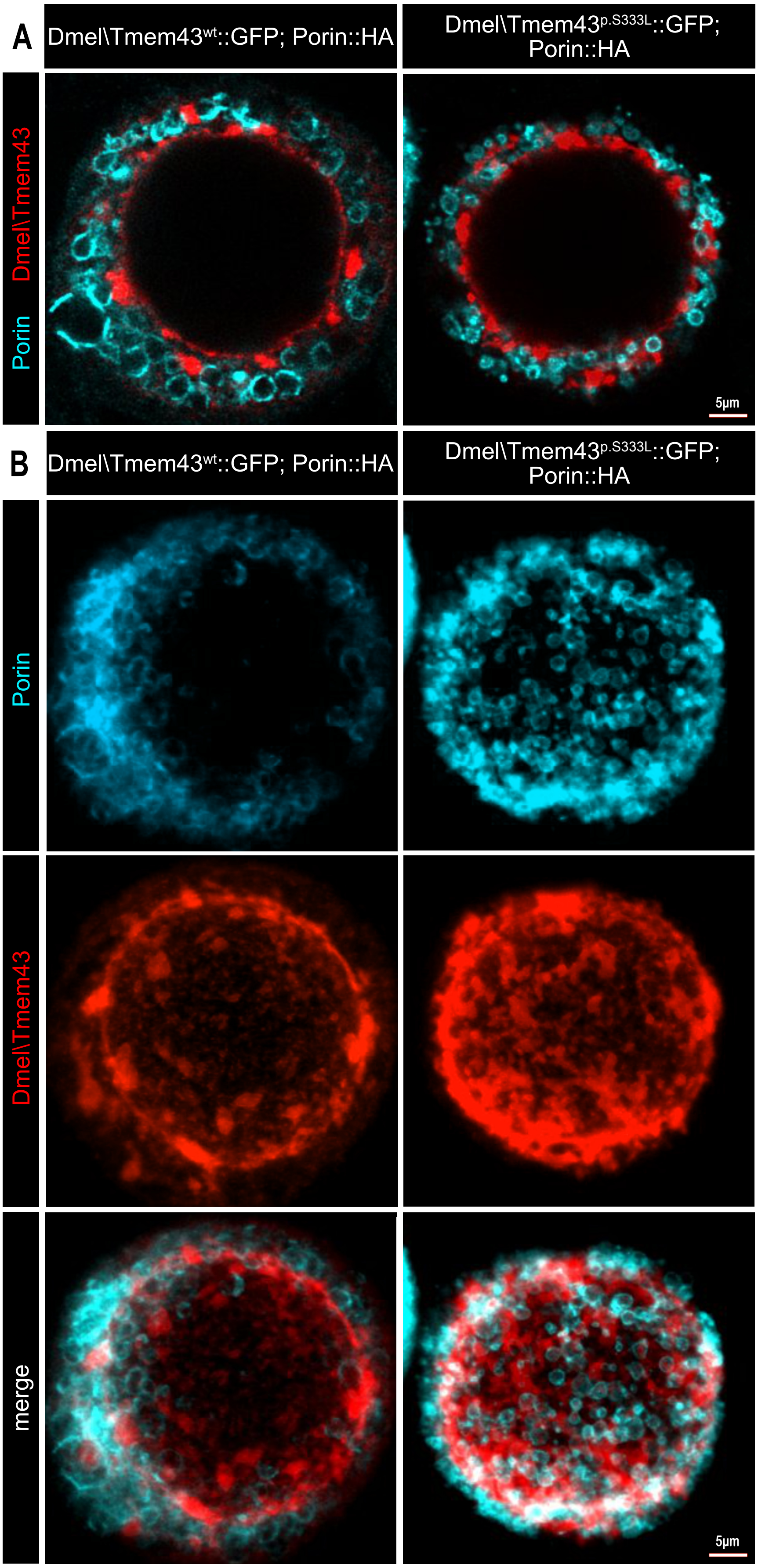

Supplementary Figure 3: Representative images of *Sf*21 cells co-expressing Dmel\Tmem43wt::GFP or Dmel\Tmem43p.S333L::GFP with Porin::HA. (A) Optical slices stained for Dmel\Tmem43wt::GFP (red channel) and Porin::HA (cyan channel). (B) Average intensity projections of the same cells with individual and merged channels.

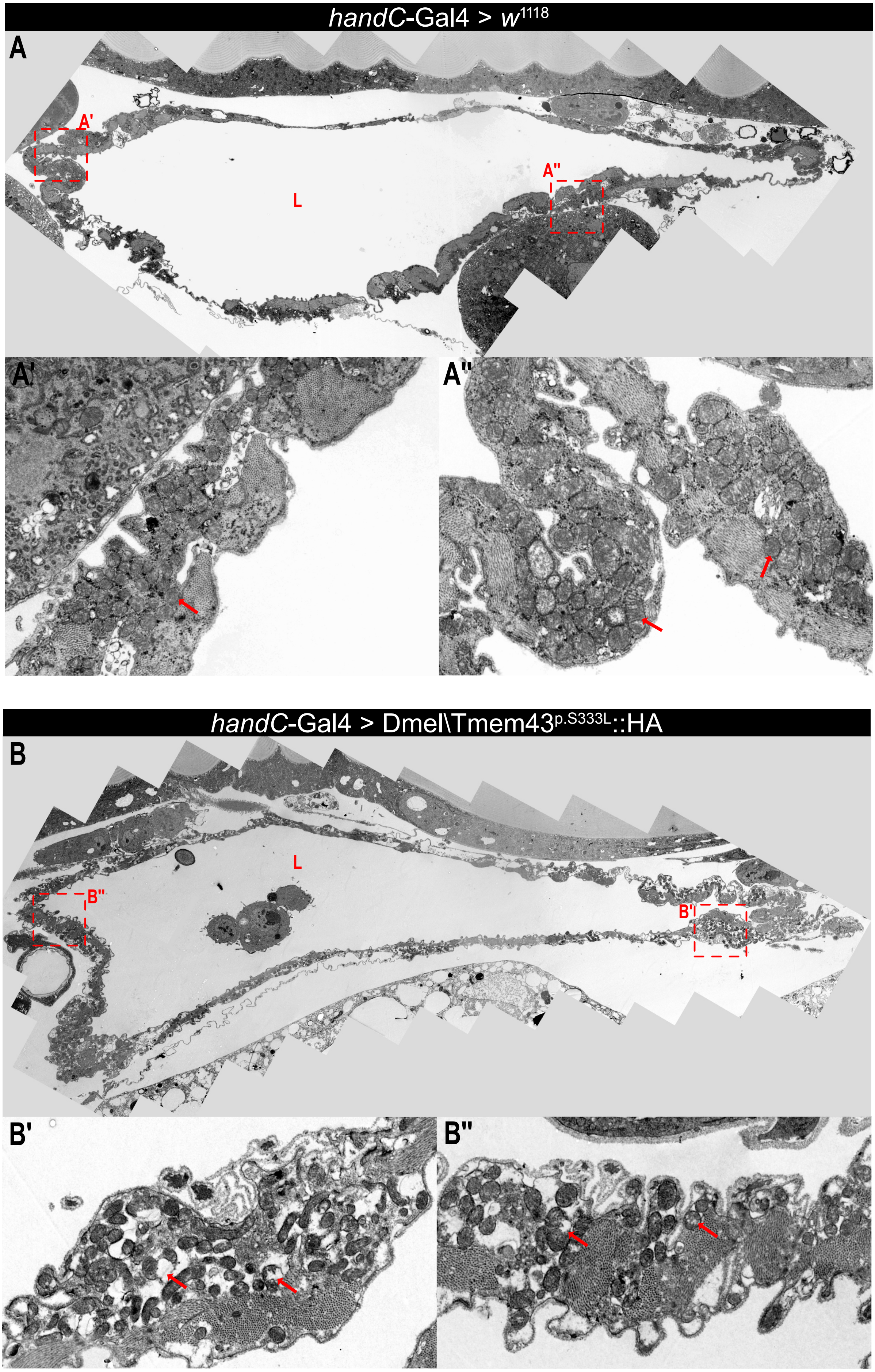

Supplementary Figure 4: (A) Representative transmission electron micrograph of a cross-sectioned heart from a 3rd instar larva expressing *w*1118 in a heart-specific manner (*handC*-Gal4). A´ and A´´ show high-magnification images of cardiomyocytes with intact mitochondria (arrows). (B) Representative transmission electron micrograph of a cross-sectioned heart from a 3rd instar larva expressing Dmel\Tmem43p.S333L::HA in a heart-specific manner (*handC*-Gal4). B´ and B´´ show high-magnification images of cardiomyocytes with numerous damaged mitochondria (arrows).

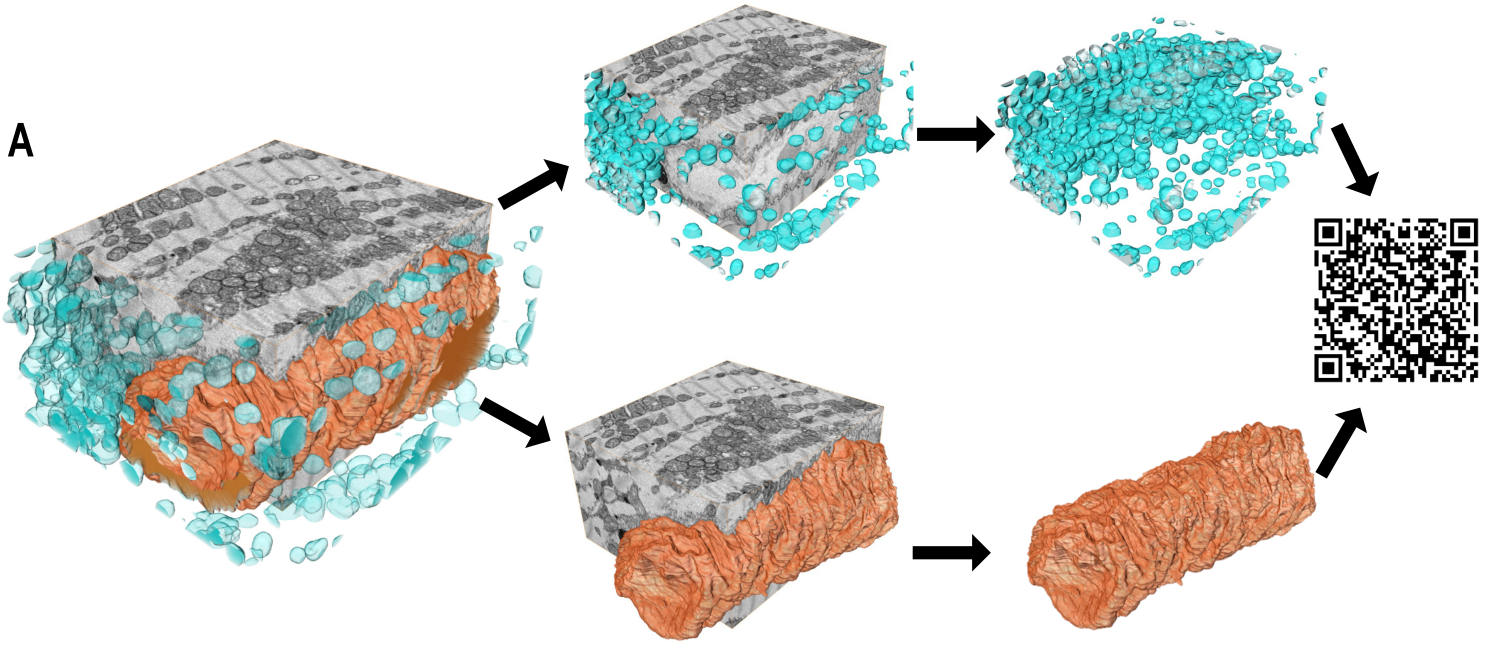

Supplementary Figure 5: (A) The figure illustrates the segmentation of mitochondria and nuclei based on serial block-face SEM datasets. Mitochondria are coloured in blue, and the nucleus is coloured in orange. The QR code links to movies that depict the individual three-dimensional datasets.
